# Survey of Localizing Gradient Damage in Static and Dynamic Tension of Concrete

**DOI:** 10.3390/ma15051875

**Published:** 2022-03-02

**Authors:** Adam Wosatko

**Affiliations:** Faculty of Civil Engineering, Cracow University of Technology, Warszawska 24, 31-155 Cracow, Poland; adam.wosatko@pk.edu.pl; Tel.: +48-12-628-2561

**Keywords:** localizing gradient damage, gradient activity function, tension, concrete cracking, impact load, dynamics, finite element method

## Abstract

The continuum damage model should be regularized to ensure mesh-insensitive results in simulations of strain localization, e.g., for concrete cracking under tension. The paper confronts the conventional gradient damage model with its upgrade including a variable internal length scale. In these models, the Helmholtz free energy depends additionally on an averaged strain measure and its gradient. In the formulation for dynamics the equations of motion are discretized simultaneously with an averaging equation. If gradient regularization is employed with a constant internal length parameter, then an artificially expanded damage zone can occur in the strain softening analysis. This broadening effect can be inhibited by a gradient activity function. The localizing character of the gradient activity has physical motivation—the nonlocal interactions in the fracture zone are reduced with the damage growth. The internal length can decrease exponentially or as a cosine function. After presentation of the theory, including the free energy definition, the finite element analyses of three different examples connected with tensile cracking in concrete are discussed: static tension of a double-edge-notched specimen, dynamic direct tension for a configuration without or with a reinforcing bar and tension of an L-shaped specimen under static and dynamic loading.

## 1. Introduction

Continuum damage mechanics in the most basic version [1] introduces the idea of scalar damage measure reducing the elastic stiffness. A modelling of softening in quasi-brittle materials such as concrete without any regularization leads to results dependent on the introduced discretization. The (initial) boundary value problem—(I)BVP becomes ill-posed when the onset of strain localization occurs in the analysis, cf. [2]. In the finite element method (FEM) a simulation of cracking in concrete is represented by the localization zone limited to a band of one-element width; hence, the density of finite element (FE) mesh erroneously decides about the numerical solution. This deficiency can be partly overcome if the FE size is connected with a certain width derived from the fracture energy, see [3,4]. A regularization should be taken into account in proper modelling of composites, especially of quasi-brittle materials as concrete. There are many concepts to make the concrete model regularized, but in this paper a higher-order theory including a gradient term is employed, according to the fundamentals given in [5].

The scalar damage model with a gradient enhancement was first proposed in [6]. Based on [7,8], the Helmholtz free energy for the damage model with the presence of averaged strain measure is shown in the paper. In the formulation for dynamics an extra averaging equation is added to the equation of motion. The gradient damage model after discretization has independent interpolations of the displacement and averaged strain fields. The gradient activity related to the internal length scale influences the zone of nonlocal interactions, i.e., the width of the crack band is not governed by the density of the FE mesh. It is proved that this model in the implicit version for static as well as dynamic problems (i.e., for wave propagation problems) is truly nonlocal (see [9]). A wide overview of gradient-enhanced and other nonlocal models for concrete is performed in [10,11].

When the internal length scale in the conventional gradient damage model (CGD) is assumed to be constant as, e.g., in [6,12], then the issue of an artificially expanded damage zone can occur. In fact, the intensity of averaging is the same during the whole localization process and it induces nonlocal mapping of the active damage zone into its enlarged neighbourhood. This shortcoming of the CGD model was first observed by Geers [13,14]. Gradient models can be upgraded by the so-called over-nonlocal formulation presented, e.g., in [15,16]. This approach comes from [17] and it is applied over the years in integral nonlocal models (see, e.g., [18,19,20]). A linear combination of local and nonlocal state variable (e.g., equivalent strain measure) is used and, moreover, its proportion may change during the loading history, cf. [21,22]. Another upgrade is suggested in [23], where the internal length scale is not represented by a scalar variable, but using a second order tensor as a function of principal stresses at a material point. The idea is known from the CGD model, where in two dimensions the region of averaging determined by a circle transforms into an area specified by an ellipse oriented according to the principal stresses. When the directions of nonlocality are distinguished that way, the modelling of the localization zone becomes anisotropic. Some modification of this approach is the so-called smoothing gradient damage model, where the averaging region depends additionally on a coefficient related to the equivalent strain and smoothly decreasing interaction [24,25].

**Figure 1:**
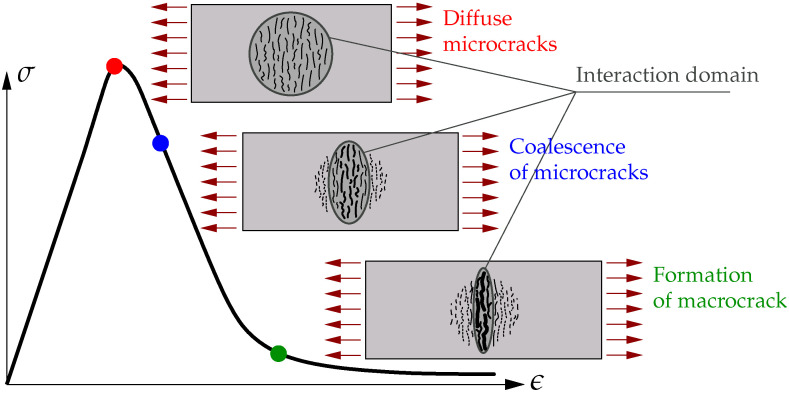
Idea of localizing interaction domain for microcracks in tensile specimen.

The constant value of the internal length scale can be replaced by a function of gradient activity. It was first proposed in [13,14], where apart from the averaging equation, one more extra continuity equation for damage or gradient activity variable was introduced. It means that the formulation includes three fields and additional degrees of freedom are present in the finite element. The third field is interpolated to stabilize the iteration process during the computations. This approach can be called the transient gradient damage model (TGD) and it can be modified according to [26]. The gradient activity function is shifted to the denominator in the averaging equation and because of that two primary fields are preserved in the FE interpolation. In [26], the gradient activity increases with the equivalent strain. Another concept is the so-called localizing gradient damage model (LGD) originated in [27,28], where the damage zone is controlled through a reduction of the gradient activity and at the same time the averaging region. An overview of different damage formulations with constant or variable internal lengths, based on the benchmark of one-dimensional tensile bar, is shown in [29]. A comparison and generalization of TGD and LGD models is widely discussed in [30]. Based on [27,31], Figure 1 depicts the idea of a change of the interaction domain in the specimen under uniaxial tension. It is seen that the averaging region narrows when diffuse microcracks progress to the formation of a macrocrack. The decreasing function of gradient activity seems to be more physical, i.e., the influence of nonlocality should be reduced together with the increase of damage.

The averaging equation given in [27,28] has a different form from the one commonly known and written originally in [6]. The gradient operator is also applied to the function of evolving length scale, not only to the averaged strain. The LGD model has been intensively explored in recent years. Extensive research of this model is presented in [31], also in the context of verification using different examples, not only for concrete, but also for other composite materials. As shown in [32], the model is able to reproduce the size effect. The Ottosen equivalent strain measure as an alternative loading function [33] can be applied in the LGD model to simulate properly a mixed-mode concrete cracking. A so-called micro inertia effect can be considered in the formulation to analyze cracking in dynamic problems (see [34,35]). Moreover, the LGD model can be used in an advanced multi-field analysis [36], where the mechanical problem is coupled to water transport and thermal problems. Different methods of mesh adaptation for this model are suggested in [37].

In this paper, a formulation for dynamics, but without the micro inertia effect, is taken into account. The results for the CGD and LGD models are confronted. Both of them are implemented by the author in the FEAP package [38]. When the LGD model is used, two different functions can decide about the decrease of the gradient activity. The first one has the exponential character and it is known from [27,28], while the second one changes according to the cosine function.

In the paper, the finite element (FE) analysis focuses on the modelling of the cracking phenomenon in concrete for tension tests. Based on the experiments of uniaxial tension for different composite materials, not only the tensile strength can be estimated, but also the fracture energy when the post-peak response is observed. Direct tension can be experimentally investigated using symmetric specimens with two rectangular (see, e.g., [39]) or triangular (see, e.g., [40]) notches as well as dog-bone shaped specimens (see, e.g., [41]). Typically, specimens in the experiments are gripped on opposite flat sides and pulled on one or both sides. Mode I fracture is obtained. It is also possible to examine concrete cracking using large-scale specimens as for example in [42]. However, it is known that the size effect can be validated using direct tension tests, cf. [41]. Another type of a experimental test is compact tension of a composite specimen, e.g., [14,43]. The first benchmark in the current paper is a double-edge-notched specimen under direct static tension according to [39]. In addition, based on the numerical analysis presented in [44], a study of the LGD model is performed for a dynamic direct tension test of plain and reinforced concrete bar. A slightly different investigation refers to fracture in an L-shaped concrete specimen. The final example presented in this paper for the CGD and LGD models is based on the experiment in [45]. A similar experimental study of L-specimen under different loading rates is shown in [46]. The induced tension in the L-shaped specimen is still direct, but the character of failure can evolve from mode I to mixed mode. Next, a separate group are indirect tests. The splitting test was carried out experimentally and reported by many researchers (see, e.g., [47,48,49]). The compression between the platens activates a perpendicular tension in the middle of the cylinder, hence primary and secondary cracks are generated. This experiment is able to provide the tensile strength for quasi-brittle materials. The split in the concrete cylinder under a static as well as an impact loading can be reproduced using different regularized models, cf. [44,50,51,52,53]. The tensile strength is also determined for notched or unnotched beams under three-point bending. The size effect for such beams has been examined in many papers (see, e.g., [54,55,56,57]). Quite a broad overview of experimental and numerical tension tests is described in [58].

The content of the paper is as follows. After introduction, Section 2 describes the theory connected with the gradient damage model, in sequence: consequences of postulating a gradient-dependent free energy, the formulation with its discretization and juxtaposition of functions employed in the computations. Section 3 shows three examples: static uniaxial tension of a notched bar, dynamic tension of an unnotched bar without and with reinforcement and finally static and dynamic tension of the L-shaped configuration. Section 4 and Section 5 summarize the work and the results presented in the paper.

## 2. Fundamentals of Implemented Model

### 2.1. Thermodynamic Analysis

The description of the theory starts by defining an internal variable $\bar{\epsilon }$ which is related to deformation and will turn out to be an averaged (nonlocal) strain measure. It is postulated that the Helmholtz free energy depends on this variable and its gradient as follows, cf. [7,8,27,28,59]:

(1) $$\Psi \left(\epsilon ,\bar{\epsilon },\nabla \bar{\epsilon },\omega \right)=\Psi_{\;1}\left(\epsilon ,\omega \right)+\Psi_{\;2}\left(\epsilon ,\bar{\epsilon }\right)+\Psi_{\;3}\left(\nabla \bar{\epsilon }\right)$$

Absolute tensor notation is used in this subsection. The individual components on the right side of this equation are defined as:

(2) $$\Psi_{1}\left(\epsilon ,\omega \right)=\frac{1}{2}\left(1-\omega \right)\epsilon :D^{e}:\epsilon ,\;\Psi_{2}\left(\epsilon ,\bar{\epsilon }\right)=\frac{1}{2}H{\left(\tilde{\epsilon }-\bar{\epsilon }\right)}^{2},\;\Psi_{3}\left(\nabla \bar{\epsilon }\right)=\frac{1}{2}A\nabla \bar{\epsilon }\cdot \nabla \bar{\epsilon }$$

where ϵ is the strain tensor, ω∈[0,1] is the scalar damage parameter, $D^{e}$ is the fourth order tensor of elastic stiffness, $\tilde{\epsilon }\left(\epsilon \right)$ is an equivalent strain measure, *H* is a constant and *A* is proportional to the square of an internal length scale. In [8,59] an alternative form of the free energy is written in terms of damage and its gradient. A more complex form of the Helmholtz free energy can be postulated for a coupled gradient damage-plasticity model [60].

For a nonlocal continuum formulation the use of the global form of the Clausius–Duhem dissipation inequality for isothermal processes is needed:

(3) $$\dot{D}=\int_{B}\;\left(\sigma :\dot{\epsilon }-\dot{\Psi }\right)\;dV\geq 0$$

where $\dot{D}$ denotes the time rate of dissipation and σ is the stress tensor. It is defined for a certain domain B, occupied by the material body. Next, the time derivative of Ψ is calculated:

(4) $$\dot{\Psi }=\frac{\partial \;\Psi }{\partial \;\epsilon }:\dot{\epsilon }+\frac{\partial \;\Psi }{\partial \;\bar{\epsilon }}:\dot{\bar{\epsilon }}+\frac{\partial \;\Psi }{\partial \;\nabla \bar{\epsilon }}\cdot \nabla \dot{\bar{\epsilon }}+\frac{\partial \;\Psi }{\partial \;\omega }:\dot{\omega }$$

and further:

(5) $$\frac{\partial \;\Psi }{\partial \;\epsilon }=\left(1-\omega \right)\;D^{e}:\epsilon +H\;\left(\tilde{\epsilon }-\bar{\epsilon }\right)\;s,\;\;s=\frac{\partial \;\tilde{\epsilon }}{\partial \;\epsilon }$$

(6) $$\frac{\partial \;\Psi }{\partial \;\bar{\epsilon }}=-H\;\left(\tilde{\epsilon }-\bar{\epsilon }\right),\;\;\frac{\partial \;\Psi }{\partial \;\nabla \bar{\epsilon }}=A\nabla \bar{\epsilon },\;\;\frac{\partial \;\Psi }{\partial \;\omega }=-\frac{1}{2}\;\epsilon :D^{e}:\epsilon =-Y$$

where *Y* is the damage energy release rate. Substituting Equation (4) into inequality (3) gives:

(7) $$\dot{D}=\int_{B}\;\left[\left(\sigma -\frac{\partial \;\Psi }{\partial \;\epsilon }\right):\dot{\epsilon }-\frac{\partial \;\Psi }{\partial \;\bar{\epsilon }}:\dot{\bar{\epsilon }}-\frac{\partial \;\Psi }{\partial \;\nabla \bar{\epsilon }}\cdot \nabla \dot{\bar{\epsilon }}-\frac{\partial \;\Psi }{\partial \;\omega }\dot{\omega }\right]\;dV\geq 0$$

The first term provides the definition of stress:

(8) $$\sigma =\frac{\partial \;\Psi }{\partial \;\epsilon }=\left(1-\omega \right)\;D^{e}:\epsilon +H\;\left(\tilde{\epsilon }-\bar{\epsilon }\right)\;s$$

and, to retrieve the classical form of σ, it has to be assumed that the second component of the above definition is very small in comparison with the first one. This is obvious for elasticity (H≪E, *E* is Young’s modulus) and doubtful close to failure when ω→1, but this term is consequently neglected. Upon substitution of Equations (6) and (8) into inequality (7) it reads:

(9) $$\dot{D}=\int_{B}\;\left[H\;\left(\tilde{\epsilon }-\bar{\epsilon }\right)\;\dot{\bar{\epsilon }}-A\nabla \bar{\epsilon }\cdot \nabla \dot{\bar{\epsilon }}+Y\;\dot{\omega }\right]\;dV\geq 0$$

Next, the second term is integrated by parts:

(10) $$\int_{B}-A\nabla \bar{\epsilon }\cdot \nabla \dot{\bar{\epsilon }}\;dV=\int_{B}\nabla \left(A\nabla \bar{\epsilon }\right)\dot{\bar{\epsilon }}\;dV-\int_{\partial B}A\nabla \bar{\epsilon }\cdot N\dot{\bar{\epsilon }}\;dS$$

where N is the normal to the domain surface ∂B. As noted in [7] the formulation is in fact nonlocal already in the elastic state, since if it is assumed there is no damage growth (i.e., $\dot{\omega }=0$) and the dissipation must be equal to zero, then:

(11) $$\dot{D}=\int_{B}\;\left[H\;\left(\tilde{\epsilon }-\bar{\epsilon }\right)+\nabla \left(A\nabla \bar{\epsilon }\right)\right]\;\dot{\bar{\epsilon }}\;dV-\int_{\partial B}A\nabla \bar{\epsilon }\cdot N\dot{\bar{\epsilon }}\;dS=0$$

The sufficient conditions for Equation (11) to hold are the following equations:

(12) $$H\;\left(\tilde{\epsilon }-\bar{\epsilon }\right)+\nabla \left(A\nabla \bar{\epsilon }\right)=0\;\;\mathrm{in}\;V$$

(13) $$\nabla \bar{\epsilon }\cdot N=0\;\;\mathrm{on}\;S$$

Assuming H>0 all terms in Equation (12) can be divided by *H*. Therefore, a gradient scaling factor φ=A/H can be introduced to obtain the averaging equation for the CGD model in the following form:

(14) $$\bar{\epsilon }-\nabla \left(\varphi \nabla \bar{\epsilon }\right)=\tilde{\epsilon }$$

When damage grows (i.e., $\dot{\omega }>0$), the dissipation is:

(15) $$\dot{D}=\int_{B}Y\;\dot{\omega }\;dV>0$$

which proves the second law of thermodynamics is satisfied. It is also pointed out that in [35,61] an interpretation of the model as a special case of two-scale micromorphic gradient-enhanced continuum is provided, where Equation (14) couples macro- and micromorphic variables.

Next, the case when the gradient activity function depends on damage is taken into account, i.e., A=A(ω). The Helmholtz free energy becomes:

(16) $$\Psi \left(\epsilon ,\bar{\epsilon },\nabla \bar{\epsilon },\omega \right)=\Psi_{\;1}+\Psi_{\;2}+\frac{1}{2}\;A\left(\omega \right)\;\nabla \bar{\epsilon }\cdot \nabla \bar{\epsilon }$$

so that:

(17) $$\frac{\partial \;\Psi }{\partial \;\omega }=\frac{\partial \;\Psi_{\;1}}{\partial \;\omega }+\frac{1}{2}\;\frac{dA}{d\omega }\;{∥\nabla \bar{\epsilon }∥}^{2}$$

and the gradient norm now influences the dissipation:

(18) $$\dot{D}=\int_{B}(Y-\frac{1}{2}\;\frac{dA}{d\omega }\;∥\nabla \bar{\epsilon }{∥}^{2})\;\dot{\omega }\;dV>0$$

This inequality is satisfied provided that:

(19) $$\frac{dA}{d\omega }\leq \frac{2\;Y}{∥\nabla \bar{\epsilon }{∥}^{2}}$$

The averaging equation for the LGD model is as follows:

(20) $$\bar{\epsilon }-\nabla \left(\varphi \left(\omega \right)\nabla \bar{\epsilon }\right)=\tilde{\epsilon }$$

while Equation (13) holds.

In the conventional gradient-enhanced damage model one assumes the loading function which satisfies:

(21) $$F=\bar{\epsilon }-\kappa^{d}\leq 0,\;\dot{\kappa^{d}}\geq 0,\;F\;\dot{\kappa^{d}}=0$$

where $\kappa^{d}=\max\left(\kappa_{o},\bar{\epsilon }\right)$ and $\kappa_{o}$ is the damage threshold. Damage ω is a function of the history variable $\kappa^{d}$ and hence for the active process ω is a function of $\bar{\epsilon }$. Then the Helmholtz free energy depends only on ϵ, $\bar{\epsilon }$ and $\nabla \bar{\epsilon }$ and one can derive:

(22) $$\frac{\partial \;\Psi }{\partial \;\bar{\epsilon }}=-Y\;\frac{d\omega }{d\bar{\epsilon }}-H\left(\tilde{\epsilon }-\bar{\epsilon }\right)+\frac{1}{2}\frac{dA}{d\omega }\;\frac{d\omega }{d\bar{\epsilon }}{∥\nabla \bar{\epsilon }∥}^{2}$$

and express the dissipation as:

(23) $$\dot{D}=\int_{B}(Y-\frac{1}{2}\;\frac{dA}{d\omega }\;∥\nabla \bar{\epsilon }{∥}^{2})\frac{d\omega }{d\bar{\epsilon }}\;\dot{\bar{\epsilon }}\;dV>0$$

which is equivalent to Equation (18).

Following [8], the potential energy functional for dynamic problems can be written as a difference between the potentials of internal and external forces:

(24) $$\Pi =\Pi_{\mathrm{int}}-\Pi_{\mathrm{ext}}=\int_{B}\Psi \;dV+\int_{B}u\cdot \rho \ddot{u}\;dV-\int_{B}u\cdot b\;dV-\int_{\partial B}u\cdot t\;dS$$

where ***u*** is the displacement vector, ***b*** is the body force vector, $\rho \ddot{u}$ defines inertia forces with the density ρ and the acceleration vector $\ddot{u}$, ***t*** is the traction vector on boundary ∂B. Minimization of the above functional leads to the weak form of the equation of motion:

(25) $$\int_{B}\delta \epsilon :\frac{\partial \Psi }{\partial \epsilon }\;dV+\int_{B}\delta u\cdot \rho \ddot{u}\;dV=\int_{B}\delta u\cdot b\;dV+\int_{\partial B}\delta u\cdot t\;dS\;\forall \;\delta u$$

On the other hand, the weak form of the averaging Equation (20) can be obtained by multiplication of this equation by a variation of the averaged strain $\delta \bar{\epsilon }$ and integration over domain B. Next, integration by parts according to Green’s formula is applied to the gradient term:

(26) $$\int_{B}\delta \bar{\epsilon }\left[\nabla (\varphi \;\nabla \bar{\epsilon })\right]\;dV=-\int_{B}\nabla \delta \bar{\epsilon }\cdot \varphi \;\nabla \bar{\epsilon }\;dV+\int_{\partial B}\delta \bar{\epsilon }\;\varphi \;\nabla \bar{\epsilon }\cdot N\;dS$$

Knowing that the homogeneous natural boundary condition (13) holds, the weak form of the averaging equation is:

(27) $$\int_{B}\delta \bar{\epsilon }\;\bar{\epsilon }\;dV+\int_{B}\nabla \delta \bar{\epsilon }\cdot \varphi \;\nabla \bar{\epsilon }\;dV=\int_{B}\delta \bar{\epsilon }\;\tilde{\epsilon }\;dV\;\forall \;\delta \bar{\epsilon }$$

Notice that Equation (27) has the same nature regardless of whether the gradient activity is constant or is a function of ω. Equations (25) and (27) are the starting point for interpolation and linearization.

### 2.2. System of Matrix Equations

Henceforth, Voigt’s notation (also called matrix-vector notation) is used. The formulation for the LGD model has two primary fields, hence independent interpolations of displacements ***u*** and of the averaged strain measure $\bar{\epsilon }$ are introduced:

(28) $$u=N\;a\;\;\mathrm{and}\;\;\bar{\epsilon }=h^{T}e$$

where ***N*** and ***h*** contain appropriate shape functions. Small strains are assumed for the (I)BVP. The secondary fields ϵ and $\nabla \bar{\epsilon }$ can be computed as:

(29) $$\epsilon =B\;a\;\;\mathrm{and}\;\;\nabla \bar{\epsilon }=g^{T}e$$

where B=LN and $g^{T}=\nabla h^{T}$. Matrix ***L*** consists of differential operators. The corresponding variations are also interpolated, respectively. Equations (25) and (27) in a discretized form are as follows:

(30) $$\delta a^{T}\int_{B}B^{T}\sigma \;dV+\delta a^{T}\int_{B}N^{T}\rho N\;\ddot{a}\;dV=\delta a^{T}\int_{B}N^{T}b\;dV+\delta a^{T}\int_{\partial B}N^{T}t\;dS$$

(31) $$\delta e\;\int_{B}h\;h^{T}e\;dV+\delta e\;\int_{B}g\;\varphi \;g^{T}e\;dV=\delta e\;\int_{B}h\;\tilde{\epsilon }\;dV$$

Tractions and body forces do not depend on deformation.

The IBVP is linearized and equilibrium has to be achieved at each time step. The detailed derivation for the LGD model can be found in [30]. It finally leads to the following system of the matrix equations for dynamic problems:

(32) $$\left[\begin{array}{cc} M_{aa} & 0\\ 0 & 0 \end{array}\right]\left[\begin{array}{c} {\ddot{a}}^{t+\Delta t}\\ {\ddot{e}}^{t+\Delta t} \end{array}\right]+\left[\begin{array}{cc} K_{aa} & K_{ae}\\ K_{ea} & K_{ee}+K_{ee}^{LGD} \end{array}\right]\left[\begin{array}{c} \Delta a\\ \Delta e \end{array}\right]=\left[\begin{array}{c} f_{ext}^{t+\Delta t}-f_{int}^{t}\\ f_{\epsilon }^{t}-f_{e}^{t} \end{array}\right]$$

The incremental nodal displacements Δa and the incremental averaged strain Δe are solved for in each time step. Equilibrium is retrieved after iterations in subsequent time steps. The consistent mass matrix is determined in a standard way:

(33) $$M_{aa}=\int_{B}N^{T}\rho N\;dV$$

Obviously, this matrix is not taken into consideration for static problems. The submatrices given in Equation (32) are defined as follows:

(34) $$\begin{array}{cccc} \; & K_{aa}=\int_{B}B^{T}\;\left(1-\omega \right)\;D\;B\;dV,\; & K_{ae}=-\int_{B}G\;B^{T}\;D\;\epsilon \;h^{T}\;dV\; & \; \end{array}$$

(35) $$\begin{array}{ccc} \; & K_{ea}=-\int_{B}h\;s^{T}B\;dV,\; & K_{ee}=\int_{B}\left(h\;h^{T}+\varphi \;g\;g^{T}\right)\;dV\; \end{array}$$

(36) $$K_{ee}^{\mathrm{LGD}}=\int_{B}\;g\;g^{T}e\;\varphi {,}_{\;\omega }\;G\;h^{T}\;dV$$

where ***D*** is the elastic stiffness matrix. Additionally, the following notation has been introduced:

(37) $$G=\frac{\partial \omega }{\partial \kappa^{d}}\;\frac{\partial \kappa^{d}}{\partial \bar{\epsilon }}\;\mathrm{and}\;\varphi {,}_{\;\omega }=\frac{\partial \varphi }{\partial \omega }$$

It should be noted that $K_{ee}^{\mathrm{LGD}}$ does not exist for the CGD model (where φ is constant). The subvectors on the right-hand side in Equation (32) are defined below, the subscript *t* is skipped:

(38) $$\begin{array}{cccc} \; & f_{\mathrm{ext}}^{t+\Delta t}=\int_{B}N^{T}b^{t+\Delta t}\;dV+\int_{\partial B}N^{T}t^{t+\Delta t}\;dS,\;\; & f_{\mathrm{int}}=\int_{B}B^{T}\sigma \;dV\; & \; \end{array}$$

(39) $$\begin{array}{cc} f_{\epsilon }=\int_{B}h\;\tilde{\epsilon }\;dV,\; & f_{e}=K_{ee}\;e \end{array}$$

### 2.3. Applied Functions

In the computations included in the paper, the equivalent strain measure is determined by the modified von Mises definition [62]:

(40) $$\tilde{\epsilon }\left(\epsilon \right)=\frac{\left(k-1\right)I_{1}^{\epsilon }}{2k(1-2\nu )}+\frac{1}{2k}\;\sqrt{{\left(\frac{\left(k-1\right)I_{1}^{\epsilon }}{1-2\nu }\right)}^{2}\;+\frac{12kJ_{2}^{\epsilon }}{{\left(1+\nu \right)}^{2}}}$$

where $k=f_{c}/f_{t}$ is the ratio of uniaxial compressive and tensile strengths, ν is Poisson’s ratio, $I_{1}^{\epsilon }$ and $J_{2}^{\epsilon }$ are the strain tensor invariants.

In the literature, there are different functions representing the damage growth (see, e.g., [13]). According to the experiment [39] uniaxial softening for tension in concrete can be approximated by an exponential function. The damage history parameter $\kappa^{d}$, after exceeding the threshold $\kappa_{o}$, causes damage ω to grow asymptotically to 1 [12,63]:

(41) $$\omega \left(\kappa^{d}\right)=1-\frac{\kappa_{o}}{\kappa^{d}}\left(1-\alpha +\alpha e^{-\eta (\kappa^{d}-\kappa_{o})}\right)$$

where the parameters η and α are respectively associated with material ductility and residual stress which tends to $\left(1-\alpha \right)E\kappa_{o}$ (in one dimension). Hence, the latter parameter prevents the complete loss of material stiffness for α<1 and makes the numerical response more stable. The former one is related to fracture energy $G_{f}$ of concrete.

The third recalled function decides about the gradient activity. Function φ=φ(ω) is able to change the averaging region during the damage process. When the CGD model [6] is considered, then the gradient activity remains constant:

(42) $$\varphi_{0}=c_{\max}>0$$

The parameter $c_{\max}$ is related to the internal length scale *l* as shown in [64], i.e., $c_{\max}=0.5\;l^{2}$. When the LGD model [27,28] is taken into account, the gradient activity is reduced together with the damage growth:

(43) $$\varphi_{1}\left(\omega \right)=c_{\max}\;\frac{(1-R)\;\exp\left(-n\;\omega \right)+R-\exp\left(-n\right)}{1-\exp\left(-n\right)}$$

where $c_{\max}$ is still the half of maximum internal length scale squared, *R* is the residual level of interaction between microprocesses within the localization band and *n* is the power which changes the intensity of the gradient activity. The character of this function is localizing, because the gradient activity can only decrease. The derivative of function $\varphi_{1}$ equals:

(44) $$\frac{\partial \varphi_{1}}{\partial \omega }=c_{\max}\;\frac{(R-1)\;n\;\exp\left(-n\;\omega \right)}{1-\exp\left(-n\right)}$$

In this paper, an alternative definition of the gradient activity function is also used. The relation φ=φ(ω) and its corresponding derivative can be determined by cosine and sine functions:

(45) $$\begin{array}{cc} \; & \varphi_{2}\left(\omega \right)=c_{\max}\;\left[0.5\;\left(\cos(\pi \omega^{n})+1\right)\left(1-R\right)+R\right] \end{array}$$

(46) $$\begin{array}{cc} \; & \frac{\partial \varphi_{2}}{\partial \omega }=0.5\;\pi \;c_{\max}\;n\;\left(R-1\right)\;\omega^{\left(n-1\right)}\;\sin\left(\pi \omega^{n}\right) \end{array}$$

**Figure 2:**
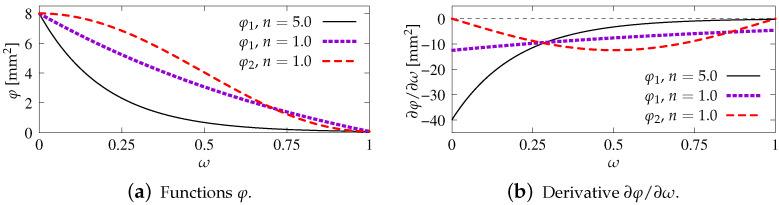
Gradient activity functions with different intensity *n*, $c_{\max}=8.0$ mm${}^{2}$, R=0.01.

The character of function $\varphi_{2}$ is also decreasing. Functions $\varphi_{1}\left(\omega \right)$ and $\varphi_{2}\left(\omega \right)$ as well as their derivatives are depicted in Figure 2. Values $c_{\max}=8.0$ mm${}^{2}$ and R=0.01 refer to first computed benchmark, discussed in the next section. The function $\varphi_{1}$ is compared for two cases of the intensity parameter, i.e., n=1.0 or n=5.0, while for $\varphi_{2}$ this is n=1.0. It is seen for all cases that non-increasing functions φ corresponds to derivatives ∂φ/∂ω which are negative or zero at most. More details on gradient activity functions can be found in [30].

## 3. Numerical Examples of Direct Tension

### 3.1. Static Tensile Cracking on Double-Edge-Notched Specimen

The first example is connected with the experiment presented in [39] for specimens with different dimensions, subjected to direct tension. In [65], the gradient plasticity model was verified using this test to show the size effect and different responses for configurations of direct tension without or with admissible rotation of the free edge. The CGD model was analyzed in [12,66], where the results for symmetric and asymmetric behaviour are obtained and the size effect is demonstrated.

**Figure 3:**
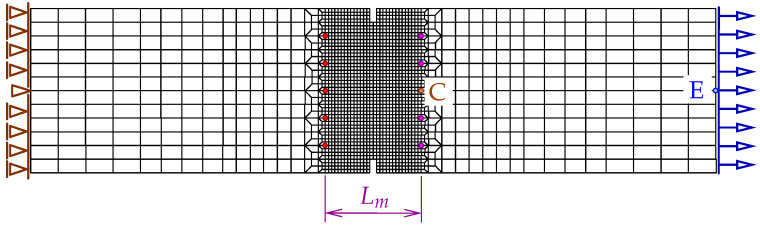
Configuration of static tension test together with mesh B, indirect displacement control at point C, elongation measured at point E.

In this paper, only a symmetric response is simulated for one selected set of dimensions. Attention is focused on the mesh-objectivity study and highlighting the difference between results for CGD and LGD models. The plain lightweight concrete bar is notched on both longer edges. The length of the specimen is L=250 mm, the height is H=60 mm, the thickness is T=50 mm. Plane stress conditions are assumed. In the numerical analysis the specimen is set horizontally, see Figure 3. Suitable boundary conditions constrain the displacements on the left, while a uniform static load acts on the right. The total elongation is measured at point E, but indirect displacement control is monitored at point C. The red points, which are adjacent to this point in one line, are linked to have the same horizontal displacement. In other words, all marked points on the right of the zone of mesh densification control the symmetric deformation of the specimen. In the computations four meshes (named A–D) with eight-noded finite elements (FEs) are employed with double densification in the middle, as depicted in Figure 3 for mesh B. Quadratic interpolation of displacements *a* and linear interpolation of averaged strain *e* together with 2×2 Gauss integration is applied in FEs. Mesh A includes 2401 nodes and 536 FEs, mesh B—7113 nodes and 1976 FEs, mesh C—13,199 nodes and 3816 FEs and mesh D—24,321 nodes and 7328 FEs. The elasticity data of the concrete model are: Young’s modulus E=18,000 MPa, Poisson’s ratio ν=0.2. The modified von Mises definition of the equivalent strain in Equation (40) is applied with the ratio k=10. The tensile strength is initially established as $f_{t}=3.4$ MPa, but actually the threshold $\kappa_{o}$ is adjusted to the maximum stress from the experiment [39] with the corresponding value captured for mesh B. In a similar way the values of parameters α and η defined in Equation (41) are fitted to reproduce the experimental diagram as close as possible. All computed cases for this benchmark are listed in Table 1. The maximum value for the gradient activity function is adopted as $c_{\max}=8.0$ mm${}^{2}$. This is the constant internal length parameter for the CGD model. The LGD model is used with the minimum level of gradient interaction R=0.01. The results for this model are compared considering functions $\varphi_{1}$ (with n=1.0 or n=5.0) and $\varphi_{2}$ (n=1.0). These functions are depicted in Figure 2a.

**Figure 4:**
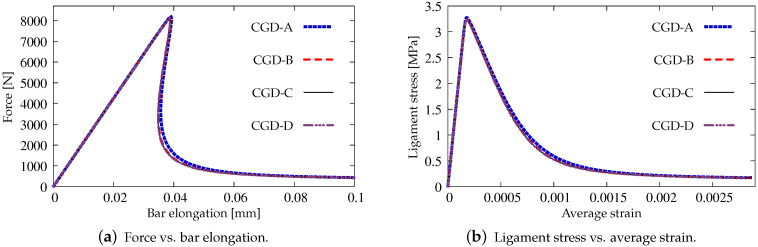
Static tension test, diagrams for CGD model, mesh-sensitivity study.

**Figure 5:**
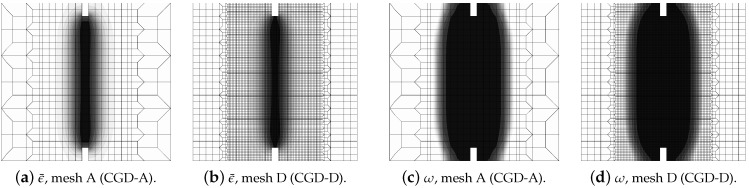
Static tension test, CGD model, distribution of averaged strain $\bar{\epsilon }$ and damage ω for two utmost cases.

Figure 4 and Figure 5 show the results for the CGD model. In Figure 4a, the diagrams of total force at the right edge versus total bar elongation measured at point E are compared for all meshes; hence, the global response is inspected. A so-called ligament stress versus average strain is plotted in Figure 4b. The concept of the ligament stress can be introduced as follows:

(47) $$\sigma_{\mathrm{lig}}=\frac{F}{B_{\mathrm{lig}}\;T}$$

where *F* is the force and $B_{\mathrm{lig}}$ is the ligament width, i.e., the width of the bar minus the depths of both notches. The average strain is the average extension of the measurement length over $L_{m}$. The measurement base $L_{m}$ (see Figure 3) is in accordance with the experiment. The placement of extensometers is distinguished by the red points. The average extension is calculated as a difference between the mean of horizontal displacements on the right (in one line with point C) and the mean of horizontal displacements observed analogically on the left. Therefore, Figure 4b presents the diagrams of nominal values. It is clearly seen that mesh-objective results are obtained. The equilibrium paths depicted in both figures overlap, however the softening branch for the coarsest mesh A marginally deviates in the middle of the descent. Figure 5 illustrates contour plots of average strain measure $\bar{\epsilon }$ and damage ω prepared for the final stage of the loading. The range of view is limited to the area in the vicinity of the notches. There are presented the results for only the two utmost cases CGD-A and CGD-D, i.e., for the coarsest mesh A and for the finest mesh D. It is confirmed that the solution is insensitive to the adopted mesh. The localization zone appears between the notches as expected. Nevertheless, a shortcoming is noticeable. The distribution of active damage in Figure 5c,d in comparison to the distribution of averaged strain in Figure 5a,b widens excessively sideways in the ligament area.

**Figure 6:**
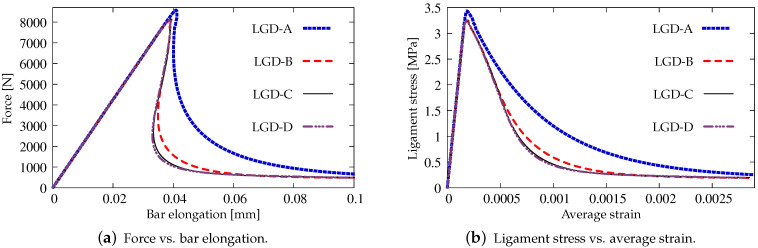
Static tension test, diagrams for LGD model using function $\varphi_{1}$ with n=5.0, mesh-sensitivity study.

**Figure 7:**
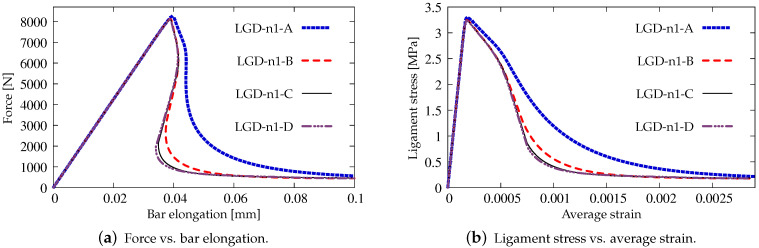
Static tension test, diagrams for LGD model using function $\varphi_{1}$ with n=1.0, mesh-sensitivity study.

Next, the results for the LGD model are presented. Figure 6 shows diagrams analogical to those presented in Figure 4, but here the LGD model with exponential function $\varphi_{1}$ and intensity n=5.0 is used. Both subfigures, with force-bar elongation diagrams as well as with ligament stress-average strain diagrams, indicate that this model seems to be mesh-dependent. The load-carrying capacity for mesh A is clearly larger than for the other three. However, together with an increasing density of the mesh, differences between subsequent diagrams vanish and finally the solutions for meshes C and D almost overlap, cf. cases LGD-C and LGD-D in Figure 6. Furthermore, in this test the snapback effect is observed and it is stronger for the solution obtained for the LGD model than for the CGD model, cf. Figure 4a and Figure 6a. Figure 7 depicts diagrams where for function $\varphi_{1}$ the intensity parameter is five times smaller, i.e., n=1.0. There are cases from LGD-n1-A to LGD-n1-D. It is visible in Figure 7a that the snapback is delayed if n=1.0. Just after the peak the equilibrium paths run down, but forward and only then backward. The convergence of solutions for subsequent denser and denser meshes has the same character as for the case when the power n=5.0.

**Figure 8:**
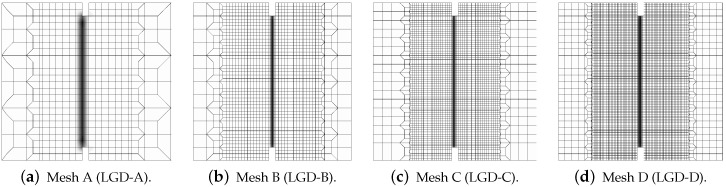
Static tension test, LGD model using function $\varphi_{1}$ with n=5.0, distribution of averaged strain $\bar{\epsilon }$, mesh-sensitivity study.

**Figure 9:**
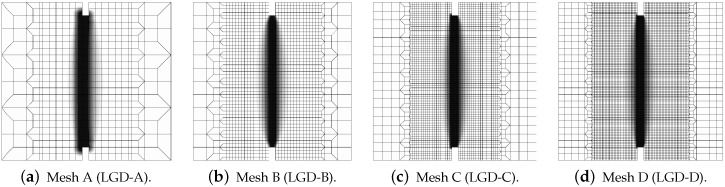
Static tension test, LGD model using function $\varphi_{1}$ with n=5.0, distribution of damage ω, mesh-sensitivity study.

**Figure 10:**
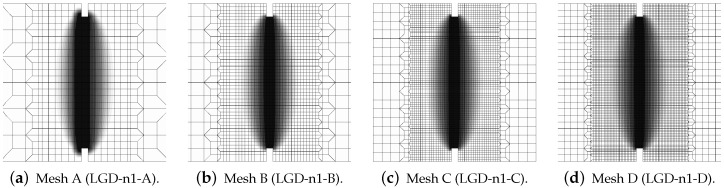
Static tension test, LGD model using function $\varphi_{1}$ with n=1.0, distribution of damage ω, mesh-sensitivity study.

The contour plots in Figure 8, Figure 9 and Figure 10 illustrate the results for the LGD model with function $\varphi_{1}$, but the distributions of damage ω in Figure 9 and Figure 10 differ if the intensity parameter n=5.0 or 1.0, analogically to the diagrams presented above. It is common that the crack is initiated near the notch. It should be noticed that the width of the notch has the width of one FE for mesh A and next it is divided into two (mesh B), three (C) or four (D) FEs along the notch width. The active localization zone for the averaged strain measure $\bar{\epsilon }$ in the case of the CGD model (see Figure 5a,b), is smeared and insensitive to the size of the notch. In reality, the shape and the size of the notch can influence the initiation point of the crack (see, e.g., experimental results in [39,40]). Moreover, due to the presence of the notches and the fact that concrete exhibits softening in the tension regime, the snapback effect is possible in this test. The solution for the LGD model is influenced by the division of the notch width. Cracking starts in the left corners for mesh A (case LGD-A) (see Figure 8a). It should be recalled that the left edge of the specimen is constrained in the analysis. For mesh B (case LGD-B) as shown in Figure 8b, the dominant averaged strain runs along the symmetry axis between the notches. For meshes C and D (cases LGD-C and LGD-D) cracking is observed along the nearest line adjacent to the symmetry axis. Hence, the solutions for meshes C and D seem to optimal in terms of energy release during the cracking process and can be recognized as mesh-objective. Of course, it can be questioned that the response depends on the division of the notch width; however, the solutions for meshes C and D are very similar. Despite the fact that the distributions of averaged strain measure $\bar{\epsilon }$ look almost the same for the LGD model with n=5.0 or 1.0, the distributions of damage ω are different, see Figure 8 and then Figure 9 and Figure 10. It is noticed that the responses for n=1.0 and meshes C and D (cases LGD-n1-C and LGD-n1-D) are almost identical, analogically to the solution with n=5.0 for meshes C and D. However, for smaller intensity n=1.0 the distribution of active damage ω is evidently wider than for n=5.0. On the other hand, it is clear that the damage zone is not spuriously broadened as for the CGD model; hence, the solution for the LGD model with $\varphi_{1}$ and the power n=1.0 is acceptable.

**Figure 11:**
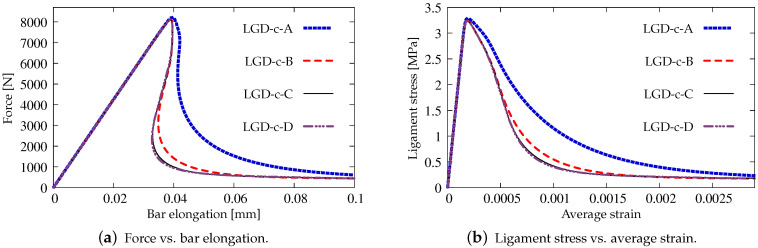
Static tension test, diagrams for LGD model using function $\varphi_{2}$, mesh-sensitivity study.

**Figure 12:**
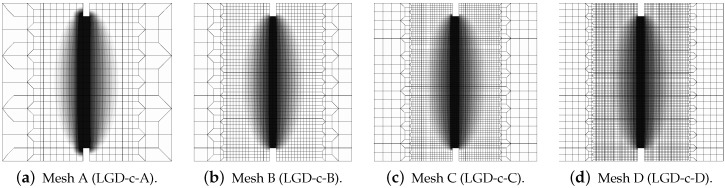
Static tension test, LGD model using function $\varphi_{2}$, distribution of damage ω, mesh-sensitivity study.

The results for the LGD model when the gradient activity decreases according to the cosine function $\varphi_{2}$ are presented separately. Again, Figure 11 depicts the force-bar elongation diagrams on the left and the ligament stress-average strain diagrams on the right. The character of all equilibrium paths is similar to the case when for the LGD model function $\varphi_{1}$ with n=1.0 is taken into account. Again, together with increasing densification of meshes, subsequent responses converge to a mesh-objective solution. Similarly, damage distributions in Figure 12 for meshes A (case LGD-c-A) and B (case LGD-c-B) differ from those obtained for meshes C and D (cases LGD-c-C and LGD-c-D). The character of the localization zone for ω when the cosine function $\varphi_{2}$ is used in the LGD model is more diffusive for smaller damage values, but finally, for the largest values of damage (ω→1.0), it reminds the distribution obtained for the exponential function $\varphi_{1}$ and n=5.0. Based on these results it can be stated that function $\varphi_{2}$ can be applied in the LGD model.

**Figure 13:**
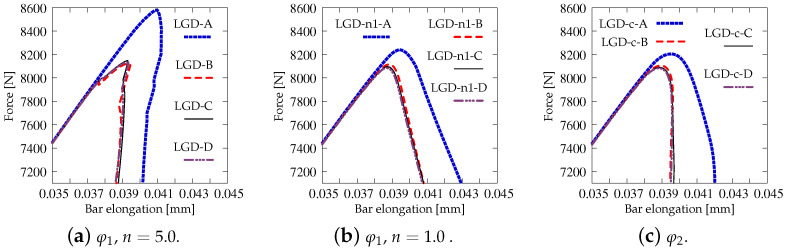
Static tension test, different options for LGD model, diagrams of force vs. bar elongation zoomed near peak.

**Figure 14:**
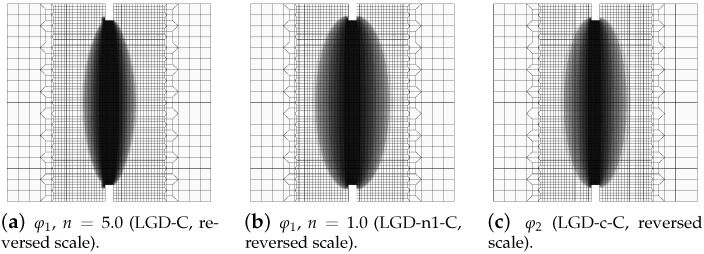
Static tension test, mesh C, distribution of gradient activity function for LGD model.

Moreover, in this test the response for the LGD model with function $\varphi_{2}$ is more stable during the iteration process. Figure 13 shows a comparison of the diagrams obtained for the total force versus the elongation at the point E, which are zoomed when the peak is attained during the loading process. When the gradient activity strongly decreases as for function $\varphi_{1}$ with the intensity parameter n=5.0, an instability in the computations for the onset of the strain localization is clearly seen. This undiserable effect is overcome for $\varphi_{1}$ and n=1.0 as shown in Figure 13b and for $\varphi_{2}$ as shown in Figure 13c. In Figure 14, the distributions of the gradient activity functions $\varphi_{1}$ and $\varphi_{2}$ are illustrated for mesh C. The scale of the values is reversed, so the black colour denotes the smallest values of function φ, which correspond to the weakest nonlocal interaction. These distributions reflect the active damage zones. The range of the gradient activity is the widest for function $\varphi_{1}$ with milder intensity n=1.0. The distribution of the gradient activity for function $\varphi_{2}$ (with the cosine) is slightly thinner. Based on this observation and taking into account the possible issue of instability for $\varphi_{1}$ with n=5.0 as indicated by the diagrams in Figure 13a, the choice of function $\varphi_{2}$ can be an effective alternative and a reasonable compromise when the LGD model is used.

**Figure 15:**
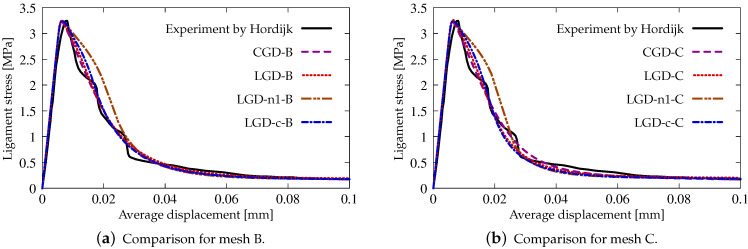
Static tension test, comparison with experiment [39], ligament stress vs average displacement, diagrams for meshes B and C.

Figure 15 presents a comparison between the applied models and with reference to the experiment [39]. The average displacement given on the horizontal axis is actually the average extension measured over the base $L_{m}$ shown in Figure 3 between marked red points and it is consistent with the measurement performed in the experiment [39]. The results in Figure 15a for mesh B and in Figure 15b for mesh C do not differ substantially, but the ones for mesh C exhibit a slightly more brittle response. Only the equilibrium paths for cases LGD-n1-B and LGD-n1-C, i.e., when the intensity parameter *n* equals 1.0, diverge from the others. In subsequent analyses, this case is no longer considered.

Summarizing the above considerations, the LGD model is more sensitive to the discretization than the CGD model. In this example, the solution for subsequent meshes approaches the final mesh-objective result. Hence, a sufficiently dense mesh should be employed in the computations. The intensity parameter *n* for exponential function $\varphi_{1}$, which decides about the rate of gradient activity for the internal length scale, should be larger than 1.0 (see also [30,31]).

### 3.2. Direct Tension Test under Impact Loading

#### 3.2.1. General Data

The second example concerns a dynamic analysis of tensile wave propagation in a concrete specimen without or with reinforcement. The results of an analogical test, but only for one discretization, were presented in [44]. There were compared two regularized models: Hoffman viscoplasticity and conventional gradient damage (CGD). A similar confrontation—gradient plasticity versus gradient damage, however using only a plain concrete bar, has been carried out in [67]. In this subsection the results obtained for the LGD model are shown for both options: plain and reinforced concrete (RC). The presentation of diagrams for the CGD model is given as a reference solution.

**Figure 16:**
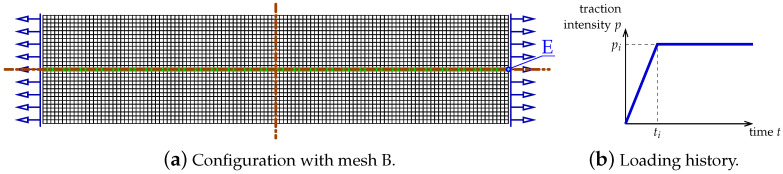
Dynamic direct tension test—definition of specimen and loading history.

The configuration of the test is illustrated in Figure 16a. This bar is supported along both symmetry axes and normal traction on both (left and right) edges is applied. The load is time-dependent according to a linear-constant function which is drawn in Figure 16b. The traction intensity $p_{i}=2.4$ MPa becomes constant for time $t_{i}=3\times 10^{-5}$ s =30μs. Each time step equals 1μs. Plane stress conditions with the thickness T=50 mm are assumed again. The length of the specimen is L=240 mm, the height is H=56 mm. This test is just to compare dynamic responses of the models; hence, *L* and *H* are adjusted to FE meshes. However, they are similar to the previous example, but the concrete bar is unnotched. Three meshes are applied. Mesh A has 2811 nodes, 960 FEs for RC configuration and the square FE size equals 4 mm. Mesh B has 10,659 nodes, 3600 FEs for RC configuration and the square FE size is 2 mm. Mesh C has 41,475 nodes, 13,920 FEs for RC configuration and the square FE size equals 1 mm. Mesh B with eight-noded FEs (and the same interpolation as previously) is depicted in Figure 16a. When reinforced concrete is taken into account, the rebar is discretized by truss elements located along the horizontal axis.

The material data for concrete are: Young’s modulus E=18,000 MPa, Poisson’s ratio ν=0.0 and density ρ=2320 kg/m${}^{3}$. Exponential damage growth function given in Equation (41) is applied with threshold $\kappa_{o}=1.8889\times 10^{-4}$ (which corresponds to $f_{t}=3.4$ MPa) and parameter α=0.99 to keep a small residual stress. Other parameters are juxtaposed in Table 2. The first column provides acronyms for the cases where plain concrete is considered in the analysis. The second column informs about acronyms for the analyses of RC models. A blank field means that only the case without reinforcement is analyzed. The equivalent strain measure is determined by the modified von Mises definition—Equation (40), k=10. In the computations two or three different values of $c_{\max}$ (connected with the maximum internal length scale) are compared. Both gradient activity functions are examined as well. The data for the steel reinforcement are: E=200,000 MPa, ν=0.0, ρ=7800 kg/m${}^{3}$ and the yield strength is $f_{y}=355$ MPa for the perfect plasticity model. Cross section $A_{r}=28$ mm${}^{2}$ indicates that the reinforcement ratio is 1%. In the case of RC, full bond between the concrete matrix and the reinforcement is adopted.

#### 3.2.2. Results for Plain Concrete

**Figure 17:**
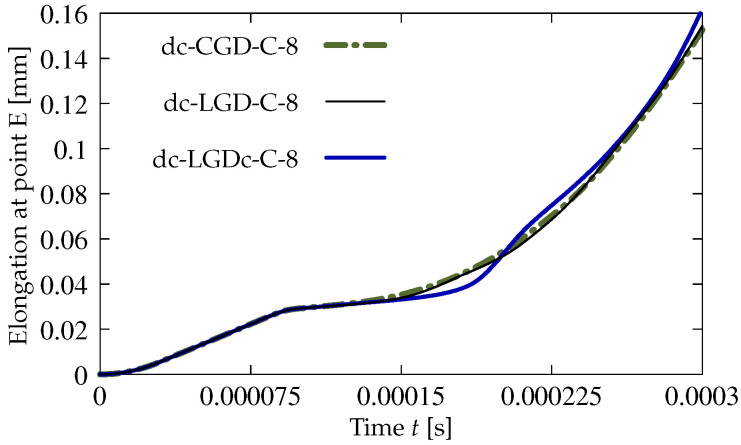
Dynamic tension test, plain concrete, mesh C, l=4 mm or $c_{\max}=8$ mm${}^{2}$, comparison of models for elongation history.

A survey of the test results commences with the comparison of CGD and LGD models based on the diagrams shown in Figure 17. The details for cases dc-CGD-C-8, dc-LGD-C-8 and dc-LGDc-C-8 are listed in Table 2. The parameters for them are selected to fit the elongation-time diagrams. In particular, this concerns parameter η. It is known, based on the comparison of CGD and LGD models for statics in [27,30] as well as in the previous benchmark, that the value of parameter η specifying the rate of damage growth should be much smaller for the LGD model than for the CGD model. This rule is also valid in the dynamic analysis, hence here η=400 for the CGD model corresponds to η=180 for the LGD model. The elongation history is monitored at point E, so the horizontal displacement is observed as a function of time. The diagrams in Figure 17 intersect each other.

**Figure 18:**
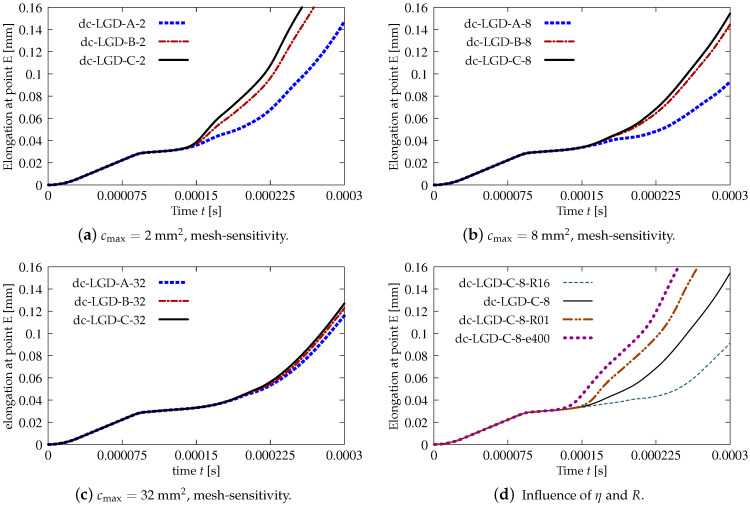
Dynamic tension test, plain concrete, elongation history for LGD model using function $\varphi_{1}$.

Figure 18 juxtaposes time-elongation diagrams for all cases solved for the dynamic direct tension test using the LGD model and exponential gradient activity function $\varphi_{1}$ (see Equation (43). It is noticed that the elongation at point E goes to infinity for all cases. It is also seen in Figure 18a,b that the results depend on the mesh, however for larger $c_{\max}$ the difference between the solutions for mesh B (case dc-LGD-B-8) and C (dc-LGD-C-8) is smaller than for $c_{\max}=2$ mm${}^{2}$. It should be explained here that the assumed value of the maximum internal length scale influences in the whole change of the gradient activity function. For example when $c_{\max}=2$ mm${}^{2}$, then value of $\varphi_{1}$ ranges to $R\times c_{\max}=0.04\times 2$ mm${}^{2}=0.08$ mm${}^{2}$ corresponding to the minimum level of nonlocal interaction, but when $c_{\max}=8$ mm${}^{2}$, then $\varphi_{1}$ approaches $R\times c_{\max}=0.32$ mm${}^{2}$. Hence for $c_{\max}=32$ mm${}^{2}$ ($R\times c_{\max}=1.28$ mm${}^{2}$) the time-elongation diagrams are very close to each other (see Figure 18c). It is observed that together with the increase of $c_{\max}$ and simultaneously with more influential gradient activity function $\varphi_{1}$ in the LGD model, the diagrams get nearest to one another, but the same level of the elongation at point E is attained slower. The parameter *R* connected with the residual level of averaging decides about the elongation rate as shown in Figure 18d. Assuming the same $c_{\max}=8$ mm${}^{2}$, the same mesh C and different values of *R* which is equal to 0.01 for case dc-LGD-C-8-R01, 0.04 for case dc-LGD-C-8 and 0.16 for dc-LGD-C-8-R16, the differences between the paths are significant. Additionally, the diagram for case dc-LGD-C-8-e400 is drawn in Figure 18d, where the parameter η equals 400 for the LGD model, exactly as the one introduced for the CGD model. The comparison presented here confirms that η should be smaller, otherwise the elongation goes to infinity the fastest of all the cases considered in this section.

**Figure 19:**
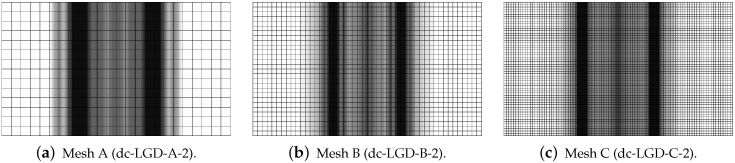
Dynamic tension test, plain concrete, LGD model using function $\varphi_{1}$ with $c_{\max}=2$ mm${}^{2}$, distribution of damage ω at t=0.0003 s, mesh-sensitivity study.

**Figure 20:**
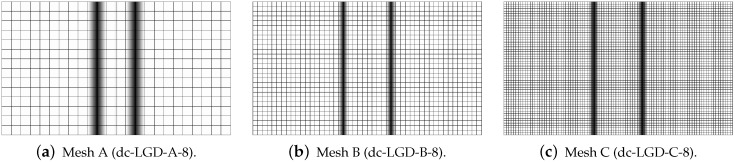
Dynamic tension test, plain concrete, LGD model using function $\varphi_{1}$ with $c_{\max}=8$ mm${}^{2}$, distribution of averaged strain $\bar{\epsilon }$ at t=0.0003 s, mesh-sensitivity study.

**Figure 21:**
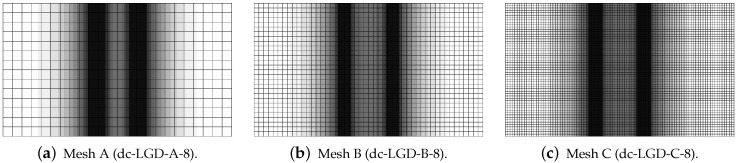
Dynamic tension test, plain concrete, LGD model using function $\varphi_{1}$ with $c_{\max}=8$ mm${}^{2}$, distribution of damage ω at t=0.0003 s, mesh-sensitivity study.

**Figure 22:**
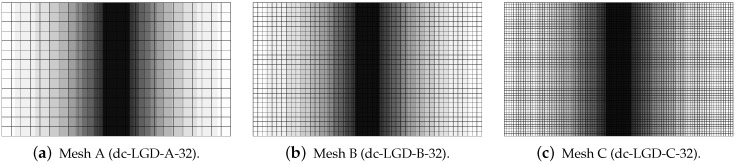
Dynamic tension test, plain concrete, LGD model using function $\varphi_{1}$ with $c_{\max}=32$ mm${}^{2}$, distribution of damage ω at t=0.0003 s, mesh-sensitivity study.

**Figure 23:**
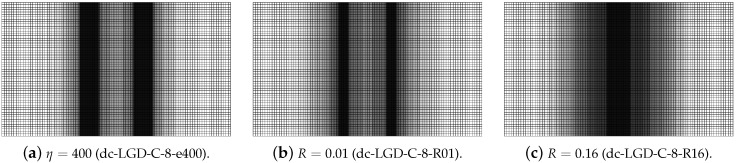
Dynamic tension test, plain concrete, LGD model using function $\varphi_{1}$ with $c_{\max}=8$ mm${}^{2}$, mesh C, distribution of damage ω at t=0.0003 s, influence of parameters η or *R*.

Figure 19, Figure 20, Figure 21, Figure 22 and Figure 23 display contour plots for the LGD model using function $\varphi_{1}$. All next contour plots in this subsection are zoomed on the same central part of the bar which is subjected to impact on the edges. Such impact loading causes formation of two waves which propagate from the sides to the center, then superpose and if only the elastic limit is exceeded for the stress, the wave stops which involves strain localization. In that case one damage zone in the middle is expected. That result is compatible with the analytical solution for the bar with strain softening, cf. [68]. Damage distributions in Figure 19 are made for cases with $c_{\max}=2$ mm${}^{2}$, subsequently for meshes A (dc-LGD-A-2), B (dc-LGD-B-2) and C (dc-LGD-C-2). The obtained responses are different. Not only two, but even three standing waves corresponding to localization zones occur (case dc-LGD-C-2 for mesh C); hence, this response results from an artificial numerical effect and the FEM analysis is mesh-dependent. When $c_{\max}$ is increased to 8 mm${}^{2}$, then one central zone of localization is anticipated based on the results for the CGD model shown in [44], but still two damage zones appear. The case of mesh A (dc-LGD-A-8) deviates from those of meshes B (dc-LGD-B-8) and C (dc-LGD-C-8). Figure 20 depicts the distribution of averaged strain measure $\bar{\epsilon }$ and Figure 21 shows damage ω at time instant t=0.0003 s. It is visible that the localization zones for mesh A are closer than for meshes B and C. In addition, it can also be noticed that the relation between $\bar{\epsilon }$ and ω is consistent with the results presented in Section 3.1—please confront Figure 8 with Figure 9 and then Figure 20 with Figure 21. The results become fully mesh-independent of the discretization for the case with $c_{\max}=32$ mm${}^{2}$ (see Figure 22). One standing wave is present in the middle of the bar for each mesh (cases dc-LGD-A-32, dc-LGD-B-32 and dc-LGD-C-32). Moreover, the width of the active damage zone is quite narrow, despite the fact that $c_{\max}=32$ mm${}^{2}$ is introduced. This value would rather be perceived as too large and causing too broad damage zone in the case of the CGD model with l=8 mm. Therefore, the gradient activity function can significantly reduce the width of the damage zone. Figure 23 includes the contour plots for the additional cases of the analysis of the direct dynamic tension test for plain concrete. It is confirmed that the parameter *R*, responsible for final nonlocal interaction, truly influences the results for the LGD model. The following cases can be investigated in a sequence: dc-LGD-C-8-R01 with R=0.01 in Figure 23b, dc-LGD-C-8 with R=0.04 in Figure 21c and dc-LGD-C-8-R16 with R=0.16 in Figure 23c. The same mesh C and $c_{\max}=8$ mm${}^{2}$ are considered. For R=0.01 and 0.04 two spurious localization zones appear. For R=0.16 one proper zone occurs due to the presence of the standing wave in the centre of the specimen, similar to the case with $c_{\max}=32$ mm${}^{2}$ and R=0.04. However, the distribution of active damage ω for R=0.16 has a more diffusive character. The above remarks coincide with the description of the elongation-time diagrams discussed in the previous paragraph, cf. Figure 18d. The contour plot of damage ω in Figure 23a for case dc-LGD-C-8-e400 with η=400 for the LGD model again exhibits two zones. The value of parameter η cannot be the same as for the CGD model. Generally, the response for the LGD model is more brittle.

**Figure 24:**
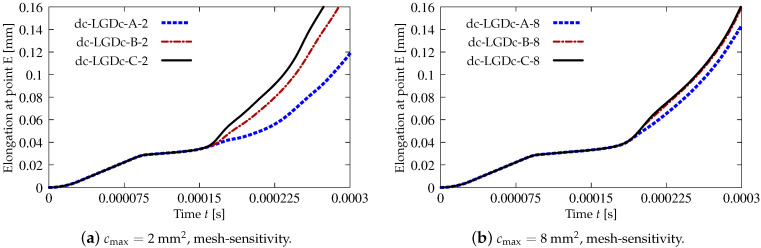
Dynamic tension test, plain concrete, elongation history for LGD model using function $\varphi_{2}$.

**Figure 25:**
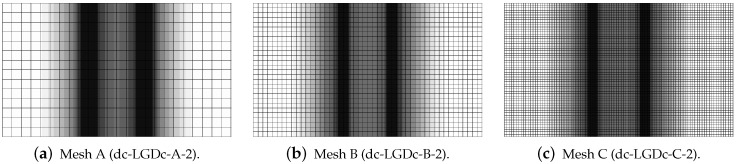
Dynamic tension test, plain concrete, LGD model using function $\varphi_{2}$ with $c_{\max}=2$ mm${}^{2}$, distribution of damage ω at t=0.0003 s, mesh-sensitivity study.

**Figure 26:**
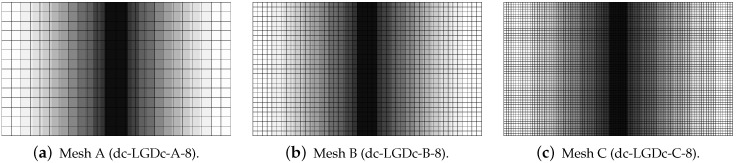
Dynamic tension test, plain concrete, LGD model using function $\varphi_{2}$ with $c_{\max}=8$ mm${}^{2}$, distribution of damage ω at t=0.0003 s, mesh-sensitivity study.

The last paragraph in this subsection describes results for the LGD model, but function $\varphi_{2}$ for variable gradient activity is introduced. This function decreases according to cosine as defined in Equation (45). Figure 24 shows the elongation at point E as the function of time. The diagrams in Figure 24a for $c_{\max}=2$ mm${}^{2}$ (cases dc-LGDc-A-2, dc-LGDc-B-2 and dc-LGDc-C-2) starting from time t≈0.00017 diverge in a slightly different directions, while the diagrams in Figure 24b for $c_{\max}=8$ mm${}^{2}$ (cases dc-LGDc-A-8, dc-LGDc-B-8 and dc-LGDc-C-8) are near to one another and only the elongation rate for mesh A is a bit smaller. It indicates that mesh-objective results can already be obtained for $c_{\max}=8$ mm${}^{2}$ when function $\varphi_{2}$ is employed for the LGD model. Damage distributions for $c_{\max}=2$ mm${}^{2}$, i.e., for dc-LGDc-A-2, dc-LGDc-B-2 and dc-LGDc-C-2 shown in Figure 25, although quite narrow damage bands are formed, are different and the width of these bands is also distinctive for each mesh. On the other hand, the increase of $c_{\max}$ to 8 mm${}^{2}$ provides very similar damage distributions as illustrated in Figure 26. One active damage zone in the middle is clearly visible. In the contrast to function $\varphi_{1}$ it can be concluded that the application of $\varphi_{2}$ in the LGD model allows one to obtain results independent of the discretization even for a smaller value of maximum internal length parameter $c_{\max}$.

#### 3.2.3. Results for Reinforced Concrete

**Figure 27:**
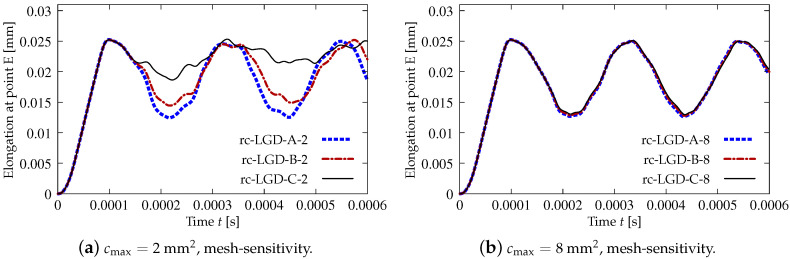
Dynamic tension test, reinforced concrete, elongation history for LGD model using function $\varphi_{1}$.

**Figure 28:**
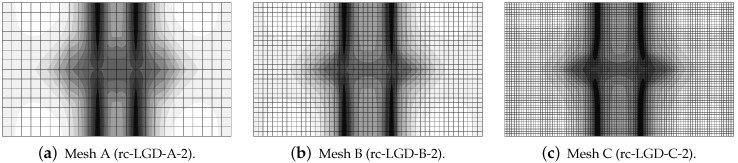
Dynamic tension test, reinforced concrete, LGD model using function $\varphi_{1}$ with $c_{\max}=2$ mm${}^{2}$, distribution of damage ω at t=0.0006 s, mesh-sensitivity study.

**Figure 29:**
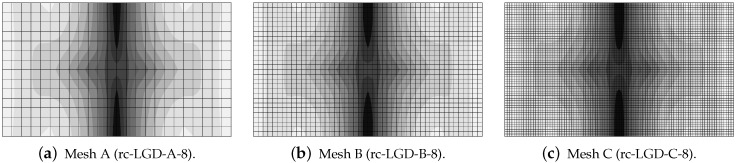
Dynamic tension test, reinforced concrete, LGD model using function $\varphi_{1}$ with $c_{\max}=8$ mm${}^{2}$, distribution of damage ω at t=0.0006 s, mesh-sensitivity study.

In this subsection the results for the RC configuration subjected to dynamic tension are presented. The solution for the LGD model with function $\varphi_{1}$ defined in Equation (43) is illustrated in Figure 27, Figure 28 and Figure 29. The elongation-time diagrams given in Figure 27 show that the presence of the rebar precludes a progress to infinite displacements. Each curve oscillates around some value of elongation. However, the diagrams for $c_{\max}=2$ mm${}^{2}$ in Figure 27a are different for each mesh. The denser the mesh is, the smaller amplitude is observed. Contour plots in Figure 28 for damage ω at the final time instant t=0.0006 s depict the localization zones placed near the centre analogically to the distributions when plain concrete specimen is considered, cf. the case with $\varphi_{1}$ and $c_{\max}=8$ mm${}^{2}$ (Figure 21) or with $\varphi_{2}$ and $c_{\max}=2$ mm${}^{2}$ (Figure 25). In the subsequent plots of Figure 28 these vertical zones slightly move away from each other. In addition, the presence of the rebar along the horizontal symmetry is seen, where damage does not activate. Actually, the most active damage is present away from the reinforcing bar. This solution is possible when full bond between the steel rebar and the concrete matrix is assumed. However, composite structures of such type can also be modelled with a representation of bond-slip by so-called interface elements, which leads to generation of many localization zones in the vicinity of the reinforcement, see, e.g., [69,70]. Moreover, it is possible to employ an interface zone called an interphase as in [71,72]. It is formed by a layer (or more layers) of FEs with non-zero thickness and represents a transition between the concrete matrix and the reinforcement as weaker concrete. The simplifying assumption of full bond as in the current computations is more suitable for modelling of RC structures with ribbed bars. The diagrams in Figure 27b for the cases with $c_{\max}=8$ mm${}^{2}$ almost overlap and curves oscillate around 0.019 mm. When the maximum internal length $c_{\max}$ is increased, then the strain localization starts from the centre points of the horizontal edges. It is observed for the distributions of damage ω in Figure 29. These cases are analogical to the results for plain concrete when $\varphi_{1}$ and $c_{\max}=32$ mm${}^{2}$ (Figure 22) or $\varphi_{2}$ and $c_{\max}=8$ mm${}^{2}$ (Figure 26) are assumed. The damage zones given in Figure 29 are quite narrow. Hence, it is shown for the RC bar under the impact loading that the LGD model with the gradient activity represented by function $\varphi_{1}$ is able to ensure the mesh-objective solution together with a proper (not too wide) distribution of active damage.

**Figure 30:**
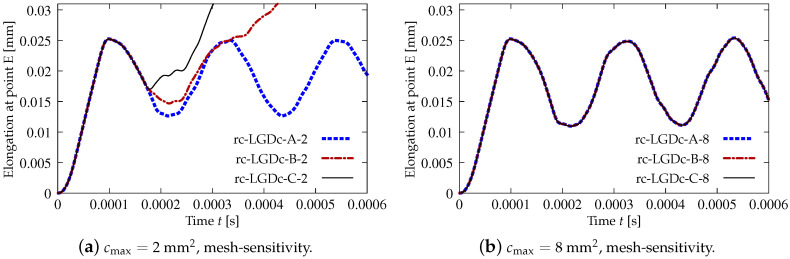
Dynamic tension test, reinforced concrete, elongation history for LGD model using function $\varphi_{2}$.

**Figure 31:**
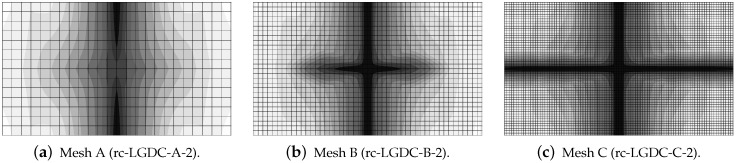
Dynamic tension test, reinforced concrete, LGD model using function $\varphi_{2}$ with $c_{\max}=2$ mm${}^{2}$, distribution of damage ω at t=0.0006 s, mesh-sensitivity study.

**Figure 32:**
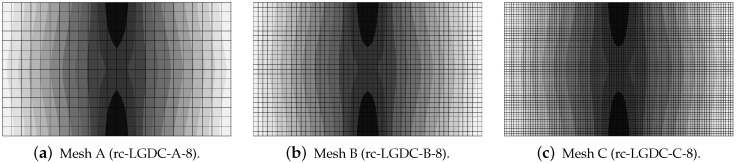
Dynamic tension test, reinforced concrete, LGD model using function $\varphi_{2}$ with $c_{\max}=8$ mm${}^{2}$, distribution of damage ω at t=0.0006 s, mesh-sensitivity study.

**Figure 33:**
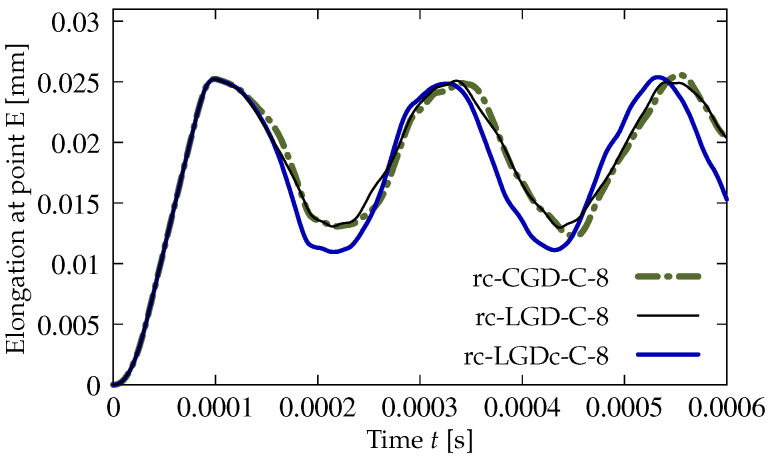
Dynamic tension test, reinforced concrete, comparison of models for elongation history, mesh C, l=4 mm or $c_{\max}=8$ mm${}^{2}$.

All previous results described in Section 3.1 and Section 3.2.2 for the LGD model with application of function $\varphi_{2}$ defined in Equation (45) constituted a reasonable alternative for the gradient activity determined by $\varphi_{1}$. The results for the case with $\varphi_{2}$ and $c_{\max}=2$ mm${}^{2}$ seem to deny this possibility. The elongation history at point E, shown in Figure 30a, strongly differs for the following meshes. In case rc-LGDc-A-2 for coarse mesh A, after the initial extension, the response oscillates around 0.02 mm. In cases rc-LGDc-B-2 for medium mesh B and rc-LGDc-C-2 for fine mesh C this horizontal displacement runs to infinity, but for the latter case the elongation is more rapid. Differences are also clearly visible for the contour plots in Figure 31. The distribution of damage ω for rc-LGDc-A-2 in Figure 31a is as expected and its character is similar to the one presented for rc-LGD-A-8 in Figure 29a. Active damage develops from the centre points of both horizontal edges. The damage plots for the next meshes, i.e., for cases rc-LGDc-B-2 and rc-LGDc-C-2 depicted in Figure 31b,c, exhibit that the solution is sensitive to the adopted discretization. Damage grows also along the reinforcing bar, which seems to be an undesirable consequence of the full bond assumption. This issue vanishes if larger $c_{\max}=8$ mm${}^{2}$ is introduced. The diagrams in Figure 30b are the same for each mesh. Again, the reinforcement in the specimen inhibits the displacements going to infinity. The contour plots for damage ω in Figure 32 are almost the same for each mesh, as well. The zones of active damage are wider than for the case with function $\varphi_{1}$ and $c_{\max}=8$ mm${}^{2}$ (cf. Figure 29), but the solution with $\varphi_{2}$ is still satisfactory. Figure 33 compares the time-elongation diagrams for the CGD and LGD models. The case rc-LGDc-C-8 differs slightly from the others. However, all the diagrams have a similar character—amplitudes have a comparable range, maximum values of elongation are visible at close time instants and the horizontal displacement at point E does not go to infinity.

### 3.3. L-Shaped Specimen under Static and Dynamic Tensile Cracking

The third example is based on the experiment described in [45]. An L-shaped concrete specimen with a fixed lower edge is subjected to tensile cracking from a corner by a vertical load originating from a pull-up clamp. In [45] this structural member is investigated also with different combinations of steel reinforcing bars or orthogonal grids, but the numerical analysis presented below focuses only on plain concrete. The response of the L-specimen can be influenced by different loading rates as shown in [73] for the numerical study with the microplane model for concrete. Furthermore, dynamic fracture of the L-shaped concrete specimen is meticulously reported in [46], where authors’ experimental tests are compared with the numerical study. The gradient-enhanced damage model linked with the microplane damage model is verified in [74] by means of a static analysis for the L-specimen. This test for statics is analyzed using the LGD model, see [31,37]. In the current paper, the numerical analysis is carried out for statics as well as for dynamics and the results for the CGD and LGD models are confronted.

**Figure 34:**
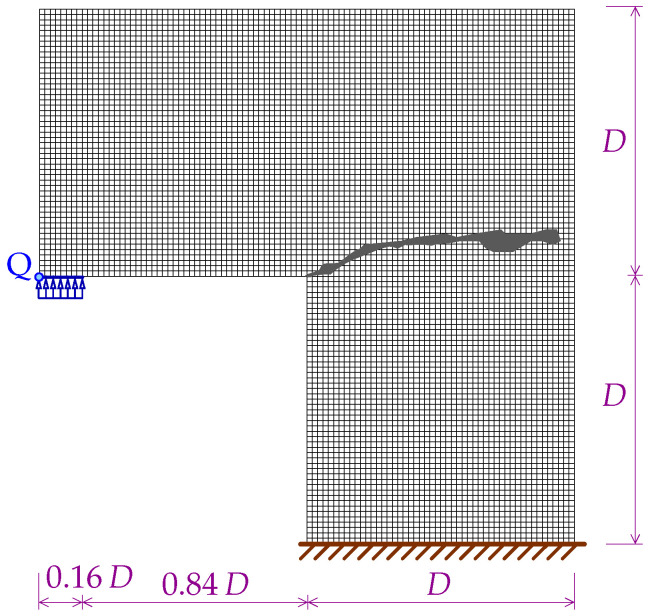
Configuration of L-shaped specimen together with mesh A and area of crack pattern for experiment with static loading performed in [45].

Figure 34 presents the L-shaped specimen together with the illustration of the fixed edge and the place where the loading is applied. The geometry of the L-specimen is determined by the characteristic size D=250 mm. The area of crack pattern for the experiment performed in [45] for plain concrete configuration is also depicted in Figure 34. In the computations three meshes are employed. The basic mesh A shown in Figure 34 is homogeneous and square FEs have the side of 5 mm. The number of eight-noded elements is 7500, the number of nodes is 23,604. The next mesh B has 16,875 square eight-noded FEs with the side of $3\frac{1}{3}$ mm for each element, 52,279 nodes and is also uniform. The third mesh C is structural and divided into some regions with rectangular and square FEs. The number of elements is 13,218. The number of nodes is 41,796. However, the region with expected cracking is most densely discretized by FEs with the element size equal to 2.5 mm. For the static analysis the Newton–Raphson method with the arc length control is used. For dynamics the standard Newmark algorithm is applied. The dynamic loading is enforced according to a linear function, but different rates are considered. The list of examined cases is given in Table 3. The loading with average rate is 10 times slower than for the case with the fast rate and 5 times faster than for the case with the slow rate.

The elastic constants are the same for CGD and LGD models: Young’s modulus E=25,850 MPa and Poisson’s ratio ν=0.18. When dynamics is analyzed the density ρ equals 2400 kg/m${}^{3}$. The plane stress configuration with thickness T=100 mm is assumed. The damage threshold $\kappa_{o}=1.0445\times 10^{-4}$ corresponds to tensile strength $f_{t}=2.7$ MPa. Exponential softening law given in Equation (41) is employed with α=0.96 and η=400 for the CGD model or with α=0.95 and η=112.5 for the LGD model. The equivalent strain measure is introduced for both models by the modified von Mises definition (40) with k=11.4815, which reflects to compressive strength $f_{c}=31.0$ MPa. The constant internal length parameter $c_{\max}$ is equal to 12.5 mm${}^{2}$ for the CGD model. The LGD model is applied with $c_{\max}$ as the half of maximum internal length scale squared. Two options are considered: the gradient activity is determined by function $\varphi_{1}$ defined in Equation (43) with R=0.01 and n=5.0 or function $\varphi_{2}$ defined in Equation (45) with the same *R* and n=1.0, cf. Figure 2.

**Figure 35:**
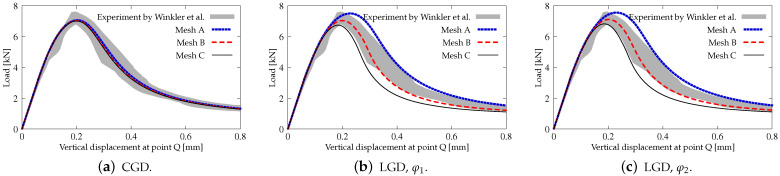
L-shaped test, statics, diagrams of load vs vertical displacement at point Q, mesh-sensitivity study.

**Figure 36:**
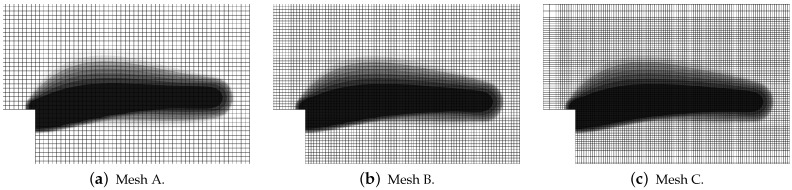
L-shaped test, statics, CGD model, distribution of damage ω, mesh-sensitivity study.

**Figure 37:**
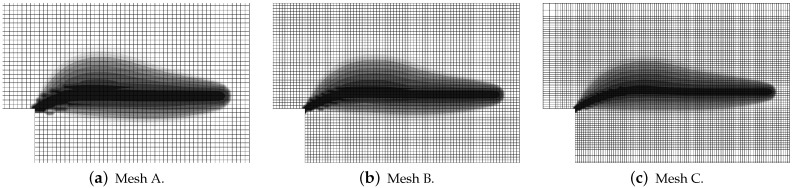
L-shaped test, statics, LGD model using function $\varphi_{1}$, distribution of damage ω, mesh-sensitivity study.

**Figure 38:**
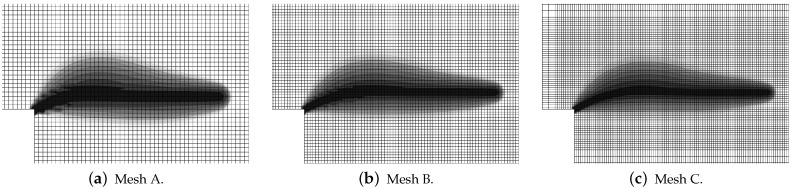
L-shaped test, statics, LGD model using function $\varphi_{2}$, distribution of damage ω, mesh-sensitivity study.

Figure 35 shows the diagrams for the load sketched in blue in Figure 34 versus the vertical displacement measured at point Q. The equilibrium paths for the CGD model in Figure 35a almost coincide and are consistent with the experimental result. It is confirmed in Figure 36, where the zones of active damage have the same shape for each mesh and coincide with the region of cracking illustrated in Figure 34. Excessive broadening of the damage zone occurs as shown in Section 3.1 for the results of the CGD model. Again, it is demonstrated that the LGD model is able to overcome this problem. Figure 37 presents analogical contour plots for damage distributions when the LGD model is used with function $\varphi_{1}$. The width of the damage zone is much narrower in the comparison to corresponding plots for the CGD model. On the other hand, a ragged area of damage occurs in a part of the localized zone starting from the corner. It is visible especially for coarse mesh A, see Figure 37a. This effect is connected with too coarse discretization for the LGD model, despite the fact that 7500 FEs is used. The LGD model demands really refined meshes in the computations, see also [27,30,31]. The problem of the zone with a non-smooth edge vanishes together with a denser mesh, cf. Figure 37b for mesh B and Figure 37c for mesh C. Moreover, the ragged areas in the damage distributions are less distinct when the LGD model with the gradient activity using the cosine function $\varphi_{2}$ is taken into account (see Figure 38). The zone of active damage for mesh C in Figure 38c has fully smooth shape and this solution resembles the cracked area from the experiment, cf. Figure 34. The load-displacement diagrams for the LGD model are depicted in Figure 35b for function $\varphi_{1}$ and in Figure 35c for function $\varphi_{2}$. They are in the limit of the gray region obtained for the experiment [45], but vary for the solutions obtained for subsequent meshes. The difference between meshes B and C is smaller for both the functions $\varphi_{1}$ and $\varphi_{2}$. It can be assumed that the density of mesh B is enough to achieve a quite objective solution. As shown for the first example in Section 3.1 the LGD model provides the results independent of the discretization.

**Figure 39:**
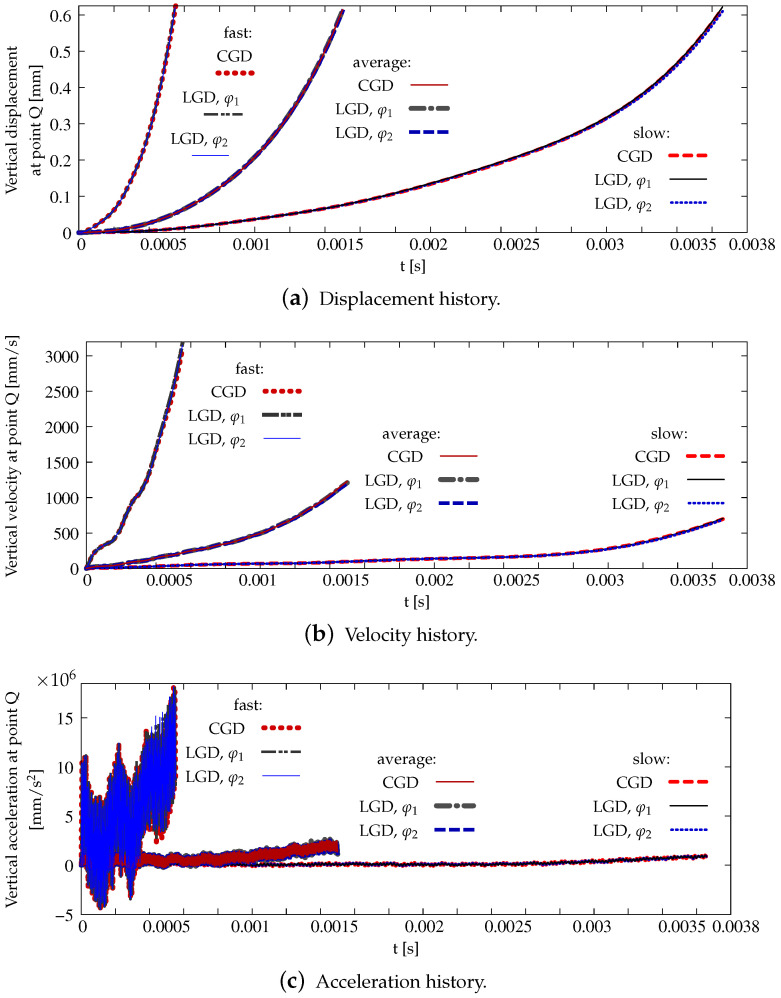
L-shaped test, dynamics, response histories, comparison of models and different rates of loading.

**Figure 40:**
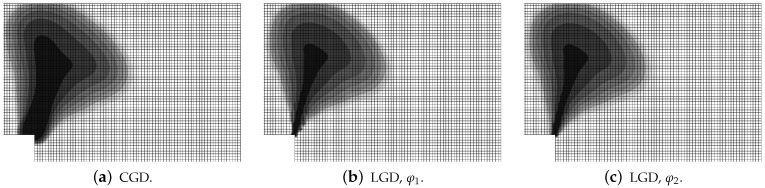
L-shaped test, dynamics, fast rate of loading, distribution of damage ω for available models.

**Figure 41:**
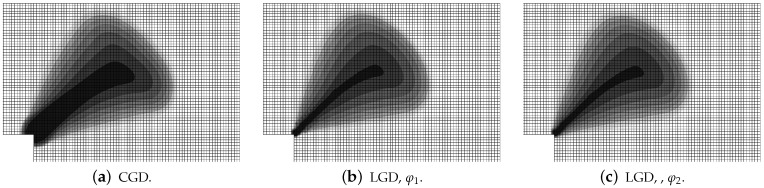
L-shaped test, dynamics, average rate of loading, distribution of damage ω for available models.

**Figure 42:**
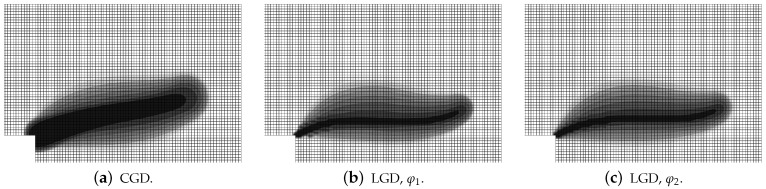
L-shaped test, dynamics, slow rate of loading, distribution of damage ω for available models.

The last part of this section is devoted to the analysis of the L-specimen subjected to the dynamic loading which grows linearly. The attention is focused on the comparison of the models, not the mesh-sensitivity study, hence only mesh B is selected in the computations. The material data for the CGD and LGD models are the same as for statics. Three cases with different rates (fast, average and slow) of the loading are analyzed, according to Table 3. The diagrams of vertical displacement or velocity or acceleration at point Q versus time for these three rates are depicted in Figure 39. It is shown that they correspond to one another for all the applied models and vary with the loading rate. In Figure 39a the displacement around 0.6 mm is attained for time of about 0.5 ms for the fast rate, approximately 1.3 ms for the average rate and close to 3.6 ms for the slow rate. The acceleration can be confronted the velocity, see Figure 39b,c. It is noticed that amplitudes of the acceleration are largest for the fast rate and the maximum value achieved are over $1.5\times 10^{7}$ mm/s${}^{2}$. They are strongly reduced to around $2.0\times 10^{6}$ mm/s${}^{2}$ for the average rate and finally the acceleration becomes very small for the slow rate. Figure 40, Figure 41 and Figure 42 show corresponding damage distributions. It is visible in Figure 40 that if the fast rate is investigated the damage zone is directed almost vertically, independently of the used model. Analogically, for the average rate damage develops diagonally for each model (see Figure 41). When the slow rate is taken into account, the damage growth in Figure 42 has a similar direction to those obtained for static computations, but at the end it goes up. The change of the fracture direction from extending upwards for the fast rate to propagating horizontally for the slow rate is also observed for the computations discussed in [46,73]. It is seen for the CGD model that the damage zone is the widest, regardless of the loading rate. For the LGD model this zone is much narrower, however the ragged areas still occur. This problem is reduced if function $\varphi_{2}$ is employed (see, e.g., Figure 41c). For cases with fast or average rates of the loading it can be distinguished that the distribution of the active damage expands and forms an elliptic area perpendicularly to the initial direction of the damage zone. It is probably connected with a transformation of mode I to mixed mode for strain localization. Moreover, branching in cracking of the concrete L-specimen investigated in the experiment can be simulated as shown in [46]. Here, the gradient-enhanced model in both implemented versions (CGD and LGD) is not able to reproduce the branching effect. This effect requires a recognition of the crack tip and for instance an extra projection method for the strain tensor [75].

## 4. Discussion

In the paper, the localizing gradient damage model (LGD) is examined in the reference to standard version of this model, called the conventional gradient damage model (CGD). The range of the study is limited to the analysis of tension tests. The results of simulations are widely discussed in Section 3. A summary of performed computations is presented in Table 4. The first part of the table shows that both versions of this nonlocal model (CGD and LGD) are considered, but the dynamic direct tension test is carried out using only the LGD model. Two different functions of the gradient activity are employed in this model. The considered specimens are subjected to static or dynamic tension. The results for double-edge-notched test are compared with the experiment performed in [39]. The second numerical test with dynamic direct tension caused by impact loading is carried out for the configuration without or with reinforcement and it is a continuation of the research published in [44]. The last example concerns tension in the L-shaped specimen, for which static or dynamic problem is solved. Computations for statics are based on the experiment presented in [45] and they are comparable with those presented in [31,74]. The results for dynamics have a rather similar character to those shown in [46]. In the analysis, three or four meshes are used in order to demonstrate a reliable mesh-sensitivity study. The same type of eight-noded FE with Serendipity shape functions for nodal displacements ***a*** and bilinear Lagrange shape functions for nodal averaged strains ***e*** as well as 2×2 Gauss integration is applied in all the computations. More details of discretization are included in Table 4 or in the description of the model for each example. Conclusions resulting from the survey described in Section 3 are listed below.

## 5. Conclusions

The paper contains the study of the localizing gradient damage model (LGD). The model [27,28] is compared with its precursor [6], i.e., the conventional gradient damage model (CGD). Both models are able to simulate cracking in quasi-brittle composite materials, in particular concrete. When the CGD model is used, a spuriously widening zone of damage occurs in simulations. This problem is overcome by means of the LGD model. The theory presentation starts from the definition of the Helmholtz free energy which depends on the strain tensor, averaged strain measure and its gradient. The averaging equation with constant or variable gradient activity is derived from this definition, cf. [7,8]. The formulation of the LGD model leads to the linearization and discretization of the (I)BVP. For dynamics the mass matrix is additionally defined, but the two-field formulation known from the CGD model holds. Both the models are implemented in the FEAP package [38]. The gradient activity in the LGD model has a localizing character, because the nonlocal interaction domain shrinks with the damage growth (see Figure 1). The gradient activity function is assumed to decrease exponentially as in [27,28] or according to a cosine function as proposed in Equation (45).

When the gradient activity function has the exponential character, then the power *n* called here the intensity parameter can affect the localizing process of nonlocal averaging significantly. In the computations usually n=5.0 is introduced, but as shown in the example of the double-edge-notched bar it can lead to convergence disturbance at the onset of strain localization, see Figure 13a. A smaller value n=1.0 causes the gradient activity to decrease slower, but then the damage zone becomes wider. In most computational cases the exponential function $\varphi_{1}$ with n=5.0 provides mesh-objective results with an appropriately narrow zone of active damage.

However, the gradient activity function $\varphi_{2}$ can be an alternative to $\varphi_{1}$. The localization zone is then more smeared for smaller damage values, but it is similar to the distribution obtained for $\varphi_{1}$ for damage values approaching 1.0. The convergence disturbance vanishes. Generally, function $\varphi_{2}$ provides correct results in the modelling of concrete cracking using the LGD model, unless a small value of $c_{\max}$ defined as the maximum internal length scale squared is applied. For instance, poor results are obtained for $c_{\max}=2$ mm${}^{2}$ in the direct dynamic tension test for the reinforced concrete (RC) configuration (see Figure 30a and Figure 31).

The application of the LGD model removes the issue of artificially broadening damage zone, but the results become more dependent on the discretization. In the paper static and dynamic tension of concrete is analyzed. Based on the results for all discussed examples, it is realized that only a reasonably refined mesh can assure a fully mesh-objective solution. As demonstrated for the L-specimen test, a problem of ragged areas in the damage distribution can occur for too coarse meshes, but it disappears upon mesh densification.

The double-edge-notched test of static tensile cracking should be computed with an extra care to keep the symmetry and proper convergence. In the dynamic direct tension test for plain concrete one zone of active damage related to a standing wave in the center is expected. The selection of values for the model parameters influences the correctness of the dynamic response. The parameter $c_{\max}$ and even more the parameter *R* associated with the residual interaction cannot be too small, because then two or more localization zones can appear. In the dynamic analysis of the L-shaped specimen, the change of direction of the damage growth zone is reproduced depending on different rates of loading, analogically to [46,73]. However, branching in concrete cracking cannot be simulated using the LGD model in this version, so in this respect it requires a further enhancement in the future.

Summarizing, the LGD model guarantees mesh-objective solution with a correct zone of active damage for static and dynamic problems, and performs better than the CGD model, but it calls for a careful selection of the values of its parameters and requires the use of denser meshes.

## Figures and Tables

**Table 1:** Computational cases for static tension test on double-edge-notched specimen (in order of appearance in figures).

Acronym	Model	Type of φ	Mesh	$\kappa_{o}$ $\times 10^{-4}$	α	η	*n*
CGD-A	CGD	$\varphi_{0}$	A	1.845	0.96	720	
CGD-B	CGD	$\varphi_{0}$	B	1.845	0.96	720	
CGD-C	CGD	$\varphi_{0}$	C	1.845	0.96	720	
CGD-D	CGD	$\varphi_{0}$	D	1.845	0.96	720	
LGD-A	LGD	$\varphi_{1}$	A	1.975	0.95	90	5.0
LGD-B	LGD	$\varphi_{1}$	B	1.975	0.95	90	5.0
LGD-C	LGD	$\varphi_{1}$	C	1.975	0.95	90	5.0
LGD-D	LGD	$\varphi_{1}$	D	1.975	0.95	90	5.0
LGD-n1-A	LGD	$\varphi_{1}$	A	1.835	0.95	100	1.0
LGD-n1-B	LGD	$\varphi_{1}$	B	1.835	0.95	100	1.0
LGD-n1-C	LGD	$\varphi_{1}$	C	1.835	0.95	100	1.0
LGD-n1-D	LGD	$\varphi_{1}$	D	1.835	0.95	100	1.0
LGD-c-A	LGD	$\varphi_{2}$	A	1.805	0.95	90	1.0
LGD-c-B	LGD	$\varphi_{2}$	B	1.805	0.95	90	1.0
LGD-c-C	LGD	$\varphi_{2}$	C	1.805	0.95	90	1.0
LGD-c-D	LGD	$\varphi_{2}$	D	1.805	0.95	90	1.0

**Table 2:** Computational cases for dynamic direct tension test.

Plain Concrete	Reinforced Concrete	Model	Type of φ	Mesh	$c_{\max}$ [mm${}^{2}$]	η	*R*	*n*
dc-CGD-C-8	rc-CGD-C-8	CGD	$\varphi_{0}$	C	8.0	400		
dc-LGD-A-2	rc-LGD-A-2	LGD	$\varphi_{1}$	A	2.0	180	0.04	5.0
dc-LGD-B-2	rc-LGD-B-2	LGD	$\varphi_{1}$	B	2.0	180	0.04	5.0
dc-LGD-C-2	rc-LGD-C-2	LGD	$\varphi_{1}$	C	2.0	180	0.04	5.0
dc-LGD-A-8	rc-LGD-A-8	LGD	$\varphi_{1}$	A	8.0	180	0.04	5.0
dc-LGD-B-8	rc-LGD-B-8	LGD	$\varphi_{1}$	B	8.0	180	0.04	5.0
dc-LGD-C-8	rc-LGD-C-8	LGD	$\varphi_{1}$	C	8.0	180	0.04	5.0
dc-LGD-A-32		LGD	$\varphi_{1}$	A	32.0	180	0.04	5.0
dc-LGD-B-32		LGD	$\varphi_{1}$	B	32.0	180	0.04	5.0
dc-LGD-C-32		LGD	$\varphi_{1}$	C	32.0	180	0.04	5.0
dc-LGD-C-8-R01		LGD	$\varphi_{1}$	C	8.0	180	0.01	5.0
dc-LGD-C-8-R16		LGD	$\varphi_{1}$	C	8.0	180	0.16	5.0
dc-LGD-C-8-e400		LGD	$\varphi_{1}$	C	8.0	400	0.04	5.0
dc-LGDc-A-2	rc-LGDc-A-2	LGD	$\varphi_{2}$	A	2.0	180	0.04	1.0
dc-LGDc-B-2	rc-LGDc-B-2	LGD	$\varphi_{2}$	B	2.0	180	0.04	1.0
dc-LGDc-C-2	rc-LGDc-C-2	LGD	$\varphi_{2}$	C	2.0	180	0.04	1.0
dc-LGDc-A-8	rc-LGDc-A-8	LGD	$\varphi_{2}$	A	8.0	180	0.04	1.0
dc-LGDc-B-8	rc-LGDc-B-8	LGD	$\varphi_{2}$	B	8.0	180	0.04	1.0
dc-LGDc-C-8	rc-LGDc-C-8	LGD	$\varphi_{2}$	C	8.0	180	0.04	1.0

**Table 3:** Cases of loading rates for L-shaped specimen.

Loading Rate	Time Step [μs]	Number of Steps	Final Time $t_{\mathrm{fin}}$ [μs]	Final Intensity $p{}_{\mathrm{fin}}$ [MPa]	Slope $p{}_{\mathrm{fin}}/t{}_{\mathrm{fin}}$ [MPa/s]
fast	4.0	150	600.0	24.0	40,000.0
average	5.0	300	1500.0	6.0	4000.0
slow	10.0	366	3660.0	2.928	800.0

**Table 4:** Summary of computed examples.

Section	Section 3.1	Section 3.2		Section 3.3	
Concrete models	CGD, LGD	LGD		CGD, LGD	
Gradient activity	$\varphi_{0}$, $\;\varphi_{1}$, $\;\varphi_{2}$	$\varphi_{1}$, $\;\varphi_{2}$		$\varphi_{0}$, $\;\varphi_{1}$, $\;\varphi_{2}$	
Specimen	double-edge-notched	unnotched		L-shaped	
Concrete	plain	plain	reinforced	plain	
Analysis	statics	dynamics		statics	dynamics
Increment	indirect displacement	standard Newmark		arc length	standard
procedure	control			control	Newmark
Loading	static	impact,		static	dynamic,
		linear-constant			linear
Number of meshes	4	3		3	
Mesh type	densified near the notches	uniform		uniform or structural	uniform
Shape	square, rectangular,	square		square,	square
of FEs	trapezoidal			rectangular	
Minimum size of FE	0.625 mm	1 mm		2.5 mm	$3\frac{1}{3}$ mm

## Data Availability

Not applicable.

## Abbreviations

The following abbreviations are used in this manuscript:

CGD	conventional gradient damage
FE	finite element
FEM	finite element method
FEs	finite elements
(I)BVP	(initial) boundary value problem
LGD	localizing gradient damage
RC	reinforced concrete
TGD	transient gradient damage
